# Narcissistic Self-Regulation and Norm Framing in Everyday Playground Encounters: Appraisal Processes in a Community-Based Experimental Study of Young Parents

**DOI:** 10.3390/ijerph23050577

**Published:** 2026-04-29

**Authors:** Avi Besser, Virgil Zeigler-Hill

**Affiliations:** 1 Department of Communication Disorders, Jerusalem Multidisciplinary College, Jerusalem 91010, Israel; 2 Department of Psychology, Oakland University, Rochester, MI 48309, USA; zeiglerh@oakland.edu

**Keywords:** narcissistic admiration, narcissistic rivalry, perceived recognition, perceived freedom threat, psychological reactance, norm framing, public parenting, playground interactions, video vignette experiment

## Abstract

**Highlights:**

**Public health relevance—How does this work relate to a public health issue?**

- Shared playground environments provide everyday community contexts in which subtle differences in norm framing shape young parents’ immediate appraisals of recognition and freedom threat.
- The study examines how routine public parenting encounters may function as micro-level psychosocial exposures that influence short-term affective, evaluative, and behavioral responses.

**Public health significance—Why is this work of significance to public health?**

- Recognition-based versus status-challenging norm framing produced marked differences in perceived recognition and perceived freedom threat, which, in turn, predicted state reactance, negative affect, evaluations of the initiating parent, and behavioral preferences.
- Contrary to the moderated mediation hypotheses, narcissistic admiration and rivalry did not moderate the indirect effects, although narcissistic rivalry, and to a lesser extent admiration, showed direct associations with reactance-related and entitlement-oriented responding.

**Public health implications—What are the key implications or messages for practitioners, policy makers and/or researchers in public health?**

- The findings identify an immediate appraisal-based mechanism through which everyday norm framing may be relevant to parenting stress in shared community settings.
- Broader implications for cumulative stress, parental well-being, and intervention design remain to be tested in longitudinal and intervention-focused research.

**Abstract:**

Everyday public parenting encounters may influence immediate stress-relevant appraisal processes. Guided by interactionist and narcissistic self-regulation frameworks, the present study examined how recognition-based versus status-challenging norm framing in a standardized playground interaction influences young parents’ immediate responses, and whether narcissistic admiration and rivalry shape these processes. A community sample of 776 Israeli parents aged 25 to 41 was randomly assigned to view one of two ultra-realistic video vignettes depicting an identical turn-taking situation framed either in recognition-based terms that emphasized fairness, shared legitimacy, and respectful coordination, or in status-challenging terms that emphasized priority claims, non-negotiability, and implied hierarchy. Participants responded from the perspective of the focal parent (i.e., a parent from the family being spoken to in the interaction). Narcissistic admiration and rivalry were assessed using the Narcissistic Admiration and Rivalry Questionnaire. Parallel moderated mediation analyses revealed that condition was strongly associated with both perceived recognition and perceived freedom threat. These appraisals, in turn, predicted state reactance, negative affect, evaluations of the initiating parent, and behavioral preferences. Recognition-based framing indirectly reduced reactance and negative affect and increased favorable evaluations through higher perceived recognition and lower perceived freedom threat. Contrary to moderated mediation predictions, narcissistic admiration and rivalry did not moderate the indirect effects. However, narcissistic rivalry, and to a lesser extent narcissistic admiration, showed consistent direct associations with reactance-related and entitlement-oriented responding. These findings identify proximal appraisal mechanisms linking subtle norm framing in public parenting contexts to immediate affective, evaluative, and behavioral reactions. More broadly, the results highlight an immediate appraisal-based process that may inform future longitudinal and intervention-focused research on parenting stress in shared community settings.

## 1. Introduction

Everyday parenting interactions in public settings often require the regulation of shared resources through subtle norm enforcement, yet individuals may differ substantially in how they appraise and respond to these cues. Young parents routinely encounter brief, commonplace exchanges in shared community spaces that can communicate either mutual recognition and fairness or hierarchy and entitlement. Although such encounters are ordinary, they may be psychologically meaningful because they shape immediate appraisals of legitimacy, autonomy, and interpersonal respect. Guided by interactionist perspectives, the present study examines whether the same situational cue is experienced differently as a function of trait-level narcissistic self-regulation, operationalized via narcissistic admiration and narcissistic rivalry [1,2,3].

### 1.1. Parenting, Everyday Stress, and a Public Health Lens

Early parenthood is a developmentally consequential period characterized by heightened daily demands and frequent role negotiations, with measurable implications for mental health and well-being. Research on parenthood and well-being indicates that parents’ functioning is shaped not only by individual characteristics, but also by the social and contextual environments in which routine parenting unfolds [4]. Consistent with this broader perspective, parental stress has increasingly been framed as a concern that is influenced by recurring daily pressures and by the environments in which parents and caregivers navigate common interactions [5].

From this perspective, everyday parenting settings are relevant because they structure repeated interpersonal experiences, including support, friction, implicit expectations, and momentary threats to autonomy or legitimacy. Longitudinal evidence suggests that contextual features of family environments are associated with parenting stress trajectories over time [6], and evidence further links parental stress to parental well-being [7]. In the present study, this broader literature serves as a contextual rationale for examining immediate appraisal and reactance processes in a routine parenting encounter, rather than as evidence that the present design directly captures cumulative stress processes.

This rationale is also consistent with research in health communication showing that autonomy-implicating cues may elicit perceived freedom threat and downstream reactance-related responses [8]. The present study extends this framework to everyday interpersonal norm enforcement in a naturalistic parenting context by focusing on immediate appraisals and short-term responses following a single exposure. Thus, the public health relevance of the study lies primarily in clarifying a proximal appraisal-based process that may be useful for future longitudinal and intervention-focused research, rather than in demonstrating broader stress or mental health effects directly.

### 1.2. Why the Playground

Public parenting environments such as playgrounds are ecologically valid micro-contexts in which recurring psychosocial exposures arise without being formally labeled as evaluation. Within these spaces, parents routinely coordinate shared resources (e.g., turn-taking at a slide), negotiate norms and expectations, and regulate children’s behavior in view of other adults and children. Ethnographic research on parent-child interactions in playgrounds documents that public space structures parental monitoring, facilitation, and interaction management, supporting the premise that these environments impose interactional demands that can become psychologically meaningful [9]. Complementing this work, recent community-level evidence suggests that children’s play spaces function not only as physical infrastructure but also as social infrastructure, facilitating neighborhood interactions associated with mental health outcomes [10].

This ecological framing is especially relevant for environmental public health research because public spaces constitute community settings in which routine psychosocial exposures can be observed and, in principle, influenced through communication and environmental design efforts. However, whether such efforts successfully shift interactional norms is likely to depend not only on the setting itself but also on how individuals interpret, adopt, and enact those norms in practice. Even brief, commonplace playground encounters may become stress-relevant events when they communicate either mutual recognition and fairness or implicit hierarchy and entitlement. Thus, identical objective interactions may be experienced as supportive or threatening depending on how they are framed and interpreted.

Consistent with this view, community research indicates that playgrounds are linked to neighborhood interaction processes and residents’ mental health, suggesting that these contexts are intertwined with broader psychosocial exposures rather than serving as a neutral backdrop [10]. Related work on public green and play spaces further indicates that such environments may structure patterns of use and social contact that are consequential for well-being [11]. Taken together, this perspective suggests that norm framing in playground interactions may function as modifiable psychosocial exposures shaping parents’ stress-relevant appraisals and responses.

### 1.3. Narcissism as Context Sensitive Self-Regulation

Contemporary theory conceptualizes narcissism not as a unitary, static trait, but as a dynamic self-regulatory system expressed in response to socially meaningful cues. The dynamic self-regulatory processing model frames narcissistic behavior as motivated self-construction, unfolding through ongoing intrapersonal and interpersonal regulation aimed at maintaining a desired self-view [3]. Within grandiose narcissism, the Narcissistic Admiration and Rivalry Conceptualization differentiates two pathways of self-regulation. Narcissistic admiration reflects an assertive self enhancement pathway oriented toward social approval and recognition, whereas narcissistic rivalry reflects a defensive status protection pathway oriented toward preventing status loss, often via antagonistic responses and derogation [1]. Recent empirical work strengthens this dynamic premise by documenting within-person fluctuations in narcissistic states across naturalistic and experimental contexts, especially in relation to perceived social feedback and status threat. This aligns well with testing subtle norm framing cues in everyday public parenting encounters rather than relying only on overt evaluative paradigms [12]. The present study focuses specifically on grandiose narcissism as operationalized by admiration and rivalry and therefore does not address vulnerable narcissism as a separate dispositional domain.

This distinction between narcissistic admiration and rivalry is critical for understanding heterogeneity in how people experience everyday social norms. Narcissistic admiration is aligned with approach-oriented motives that are sensitive to affirmation and opportunities for positive social regard. Narcissistic rivalry is aligned with defensive motives that are sensitive to cues implying reduced legitimacy, constrained autonomy, or potential status loss. These motives should be especially consequential in roles that carry strong normative expectations, such as parenting, and in public contexts where identity-relevant meanings can be inferred from brief interactions.

### 1.4. A Process Model of Status Pursuit and Domain Sensitivity

A complementary, explicitly process-oriented model clarifies why and how narcissistic reactions should depend on situational cues. The process model of narcissistic status pursuit argues that narcissistic expressions are particularly sensitive to contexts that contain status-relevant cues, including opportunities for status gain and threats of status loss [2]. These cues motivate a focus on situational appraisals as organizing mechanisms for downstream affective and behavioral responses. This process perspective is consistent with measurement work extending admiration and rivalry into domain-specific expressions, supporting the view that narcissistic self-regulation can vary across contexts and roles [13]. Relatedly, emerging evidence suggests that situation perception itself can function as a proximal trigger layer through which narcissistic traits are expressed, even when objective situational features are subtle rather than overtly evaluative [14]. This view highlights the transactional role of appraisal processes in shaping immediate self-regulatory responses and supports the use of minimally escalated, normatively framed scenarios to examine narcissistic self-regulation.

Although these models are theoretically well developed, much of the empirical research on narcissistic self-regulation has relied on explicitly evaluative laboratory paradigms or structured feedback situations. A key open question is whether narcissistic admiration and rivalry also shape reactions in everyday, identity-relevant social roles where evaluation is implicit rather than overt. Parenting represents a particularly compelling domain in this regard: it is normatively dense and frequently enacted in public settings. Even brief exchanges in community spaces can acquire self-relevance when they implicate competence, legitimacy, or respect.

### 1.5. Role Identity, Parent Identity, and Why Subtle Cues Should Matter

Identity theory offers a useful framework for understanding why parenting contexts may be psychologically consequential. Role identities are organized around meanings and standards that become salient in interaction, and discrepancies between internalized identity meanings and perceived social feedback can shape affective and behavioral responses [15,16]. Research on parental identity similarly indicates that identification with the parent role is meaningfully associated with psychological functioning and role-related processes across adulthood [17,18]. More recent evidence further strengthens this claim by showing that parental identity is empirically tied to parental well-being in specific contemporary parent populations, and that identity-related factors can contribute above and beyond demographic predictors. This reinforces the relevance of identity-consistent versus identity-threatening cues in everyday parenting contexts [19]. In public settings, identity meanings may be especially salient because behavior is observable and norm governed, and because social comparison and informal judgment are more likely to be inferred.

The present study builds on a recognition-centered framework developed in our recent work e.g., [20,21], which conceptualizes recognition as a situational appraisal of being seen, acknowledged, and treated as legitimate and respected within an interaction. Importantly, recognition is conceptually distinct from general interpersonal warmth. Whereas warmth reflects diffuse positivity or liking, recognition reflects the appraisal that one’s legitimacy, standing, and role-relevant participation are being affirmed [1,3,20,21]. Thus, recognition is not reducible to being liked. Rather, it constitutes a context-sensitive judgment that one’s status and identity are being acknowledged in a manner consistent with one’s role expectations. In identity-relevant public contexts such as parenting, this distinction is theoretically consequential because subtle cues may signal either affirmation of legitimacy or implicit diminishment of standing.

### 1.6. Psychological Reactance as an Immediate Resistance Mechanism

Psychological reactance theory conceptualizes reactance as a motivational state that arises when people perceive their freedoms are threatened or restricted, often expressed through anger and negative cognitions and linked to resistance. A foundational formulation in health communication distinguishes perceived threat to freedom from the subsequent state reactance reaction, clarifying the appraisal-to-response sequence [22]. Empirical work further supports modeling reactance as a latent construct comprised of anger and negative cognitions [23]. Recent meta-analytic evidence supports the central role of perceived freedom threat as a proximal antecedent of psychological reactance, with reliable associations with anger, negative cognitions, and resistance tendencies across communication contexts [8,24]. These findings motivate testing whether autonomy-implicating norm cues in everyday norm-enforcement encounters can similarly function as immediate triggers of reactance processes in contexts such as those examined here.

The measurement basis for state reactance is similarly well established. The Salzburger State Reactance Scale provides validation evidence supporting a three-factor structure (i.e., experience of reactance, aggressive behavioral intentions, and negative attitudes), and demonstrates convergent and divergent validity [25,26]. These frameworks translate to everyday public parenting contexts because a norm can be experienced as an illegitimate restriction on autonomy or legitimacy in a shared space, even when the interaction remains controlled and socially ordinary. Under these conditions, immediate resistance can be expressed through elevated reactance, negative affect, and preferences for actions that restore perceived autonomy or standing. Although perceived freedom threat and perceived status or legitimacy threat are conceptually related, recent communication research suggests that they are not identical. In everyday norm enforcement contexts, particularly outside explicit persuasive settings, status- or legitimacy-challenging cues may function as proximal triggers of perceived freedom threat by implying reduced agency or diminished standing in a shared space. Accordingly, the present study focuses on perceived freedom threat as the immediate appraisal underlying reactance, while recognizing that such threat may be elicited by subtly status-challenging norm framing that implies constrained agency or reduced legitimacy, rather than by overt restrictions alone [8].

### 1.7. Integrating the Frameworks: Recognition-Based Versus Status-Challenging Norms in the Playground

The core situational contrast in the present study is between two versions of the same everyday playground scenario in which two children reach a slide at the same moment. In both versions, the initiating parent articulates a norm that governs access to a shared resource. What differs is the social meaning of that norm and the way authority is enacted. In the recognition-based version, the initiating parent frames the situation around fairness, shared standards, mutual legitimacy, and explicit concern that all parties feel okay. In the status-challenging version, the initiating parent frames the same objective situation around priority claims and closure of negotiation, thereby implying implicit hierarchy and reduced legitimacy for the other family. Importantly, the other family remains silent throughout, so the cue is delivered as a one-sided norm-enforcement signal rather than a reciprocal exchange. This design isolates the initiating parent’s framing cue while preserving ecological plausibility, as brief nonresponse is a common avoidance strategy in low-level public friction. Here, the term status-challenging refers to the communicative properties of the framing cue (e.g., priority claims, closure of negotiation, implied hierarchy) rather than to a directly measured status-threat appraisal or to the exact opposite pole of perceived recognition.

The manipulation was intentionally designed to alter normative meaning rather than emotional intensity, allowing the study to test whether shifts in perceived legitimacy and autonomy, rather than overt aggression, drive appraisal processes. This manipulation was designed to function as an identity- and autonomy-relevant cue by shaping appraisals of recognition and perceived freedom, rather than as a dramatic conflict or an instance of overt aggression. The status-challenging version is controlled and normative in tone. The implied threat is conveyed through non-negotiability, spatial positioning, and a firm, closed interactional stance (e.g., reduced negotiation cues and a steady gaze), rather than increased voice volume or overt hostility. This structure allows clearer inference about how subtle, normatively framed hierarchical cues shape appraisals and immediate responses.

The proposed theoretical integration follows directly from the distinction between narcissistic admiration and rivalry. Narcissistic admiration reflects an approach-oriented focus on social approval, affirmation, and visibility. Recognition-based norms—which explicitly signal fairness, legitimacy, and mutual acknowledgment—should therefore align with admiration-related self-regulatory motives. As a result, such norms are expected to elicit higher perceived recognition and more favorable evaluations of the initiating parent. In contrast, narcissistic rivalry reflects a defensive status-protection orientation characterized by heightened sensitivity to threat and potential status loss. Status-challenging norms should therefore be more likely to evoke appraisals of perceived freedom threat, which, in turn, are expected to increase reactance-related and negative affective responses. Narcissistic rivalry is also expected to show direct associations with these outcomes and to contribute to heterogeneity in parents’ responses to identical situational cues, such that individuals higher in narcissistic rivalry exhibit stronger threat and reactance reactions. These predictions are consistent with the NARC framework and the process model of narcissistic status pursuit [1,2].

### 1.8. Extension of Prior Work and Methodological Rationale

The present study extends recognition-centered models of narcissistic self-regulation to an ecologically valid, community-based parenting context while preserving experimental control. Methodologically, we employ standardized short video vignettes that depict the same objective playground scenario across conditions, manipulating only the norm-framing language. This design isolates the focal situational cue while maintaining contextual realism, thereby enabling clean tests of trait × situation interactions within a self-regulatory framework.

This approach is consistent with established best practices for enhancing ecological validity without sacrificing internal validity. Recent methodological work provides practical guidance for designing and administering video vignettes in online studies, including steps for producing accessible, valid stimuli and documenting production decisions. This strengthens the rationale for using standardized video scenarios in the present study and enhances the transparency of the method [27]. In addition, recent reporting guidance for vignette experiments emphasizes the value of clear documentation of vignette construction and experimental manipulations, which aligns with our detailed prompt and scenario documentation strategy [28]. This perspective frames everyday public parenting encounters as potential micro-level exposures within a broader environmental health context.

#### 1.8.1. The Present Study

The present study examines whether trait-level narcissistic self-regulation plays a role in young parents’ immediate responses to recognition-based versus status-challenging norms in everyday public parenting contexts. Although the present study focuses on playground interactions as a concrete and ecologically valid context, the underlying conceptual model is not specific to playgrounds per se. Rather, it targets everyday public parenting encounters in which shared resources, implicit norms, and momentary authority claims must be negotiated in view of others. Accordingly, the playground context serves as a theoretically informative case through which broader processes of recognition, threat appraisal, and immediate self-regulatory responses in public parenting settings can be examined. The target population consisted of young parents with at least one child in the age range typical for playground use. This sampling frame is intended to reduce developmental and family structure heterogeneity while focusing on parents for whom playground-relevant routines and shared resource negotiations are especially common.

Participants viewed one standardized short video depicting a realistic playground interaction. Within participant-gender strata, assignment to the recognition-based versus status-challenging condition was randomized in a 1:1 ratio. To facilitate identification, the initiating parent’s gender was matched to the gender of the participant. As a consequence, the gender of the speaker was not analyzed as an independent factor since it was redundant with the gender of the participant. No participants in the final analytic sample identified outside the gender binary. Individual differences in narcissistic admiration and rivalry were assessed using the NARQ [1], such that the personality component of the study focused specifically on dimensions of grandiose narcissism.

#### 1.8.2. Proposed Conceptual Model

The proposed model is a conditional process framework that integrates situational norm framing with trait-level narcissistic self-regulation. Participants were randomly assigned (within gender strata) to one of two norm-framing conditions (recognition-based vs. status-challenging) and viewed a standardized playground vignette. Experimental condition was expected to influence two temporally proximal appraisals that function as parallel mediators: perceived recognition and perceived freedom threat. These appraisals capture whether the encounter is experienced as legitimizing and inclusive (recognition) or as autonomy-limiting and pressuring (freedom threat). In turn, these appraisals were expected to predict immediate responses, including state reactance, negative affect, evaluations of the initiating parent, and behavioral preferences for how the situation should be handled.

**Figure 1:**
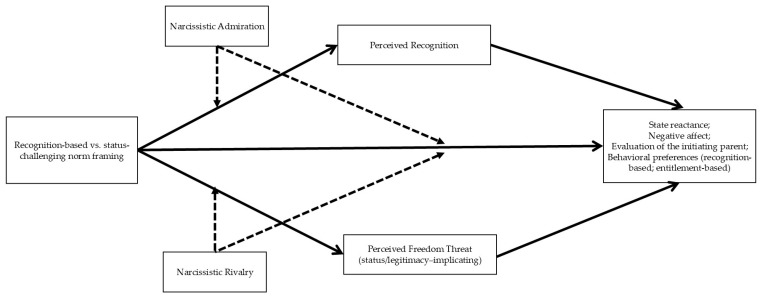
Conceptual model. Condition (recognition-based vs. status-challenging norm framing) predicts perceived recognition and perceived freedom threat (parallel mediators), which, in turn, predict the outcomes: state reactance, negative affect, evaluation of the initiating parent, recognition-based behavioral preferences, and entitlement-based behavioral preferences. Dashed arrows indicate that moderation by narcissistic admiration and narcissistic rivalry was tested for the Condition → mediator paths and for the direct Condition → outcome path. For parsimony, the main effects of admiration and rivalry that were included in the statistical models are not depicted. Solid arrows represent the hypothesized direct and mediation pathways. Dashed arrows represent the hypothesized moderating effects on the direct path and on the predictor-to-mediators paths.

Narcissistic admiration and narcissistic rivalry were modeled as continuous individual difference variables. In line with dynamic and process-oriented models of narcissism, these traits were expected to show direct associations with appraisals and responses, and they may also moderate situational effects. Consistent with Figure 1, we tested whether narcissistic admiration and rivalry moderated the situational effect of experimental condition on the appraisal layer (moderated mediation). We also tested whether experimental condition had residual direct associations with the outcomes beyond the appraisal pathways and whether these associations varied as a function of narcissistic admiration and narcissistic rivalry (conditional direct effects). Figure 1 presents the conceptual model and serves as the organizing framework for the hypotheses. These moderation tests are stated explicitly in the Section 1.8.4. below.

#### 1.8.3. Public Health Implications

From a public health perspective, the present study examines how everyday public parenting encounters in shared community spaces may shape immediate appraisal processes that are relevant to parental stress [4,5,7]. In particular, the model focuses on norm framing by other parents as a situational feature that may influence perceived recognition and perceived freedom threat, which, in turn, predict immediate affective, evaluative, and behavioral responses.

At the same time, it is important to note that the present findings do not establish broader public health effects or intervention efficacy. Rather, the goal of this was to identify potential appraisal-based mechanisms that may be relevant to future prevention-oriented and longitudinal research on parenting stress in community settings.

#### 1.8.4. Hypotheses

**H1.**  *(Condition effects on proximal appraisals). Compared to the status-challenging condition, recognition-based norm framing will elicit higher perceived recognition and lower perceived freedom threat.*

**H2.**  *(Appraisals predict immediate responses). Higher perceived recognition will be associated with lower state reactance and lower negative affect, more positive evaluations of the initiating parent, higher recognition-based behavioral preferences, and lower entitlement-based behavioral preferences. Higher perceived freedom threat will be associated with higher state reactance and higher negative affect, more negative evaluations of the initiating parent, lower recognition-based behavioral preferences, and higher entitlement-based behavioral preferences.*

**H3.**  *(Indirect effects via parallel mediators). Condition will show indirect associations with each outcome through perceived recognition and perceived freedom threat. Specifically, recognition-based (vs. status-challenging) framing will predict lower reactance and negative affect, more positive evaluations, higher recognition-based preferences, and lower entitlement-based preferences via higher perceived recognition and via lower perceived freedom threat.*

**H4.**  *(Trait main effects on appraisals and responses). Narcissistic admiration will be positively associated with perceived recognition and with more favorable immediate responses, including lower state reactance and lower negative affect, more positive evaluations of the initiating parent, higher recognition-based behavioral preferences, and lower entitlement-based behavioral preferences. Narcissistic rivalry will be positively associated with perceived freedom threat and with less favorable immediate responses, including higher state reactance and higher negative affect, more negative evaluations of the initiating parent, lower recognition-based behavioral preferences, and higher entitlement-based behavioral preferences.*

**H5a.**  *(Moderation of the appraisal layer). Narcissistic admiration and narcissistic rivalry may moderate the effect of experimental condition on the two proximal appraisals (i.e., perceived recognition and perceived freedom threat). We expected admiration to be associated with stronger differentiation between conditions on perceived recognition, whereas rivalry was expected to be associated with stronger differentiation between conditions on perceived freedom threat.*

**H5b.**  *(Conditional direct effects and situational sensitivity). Beyond the indirect effects via appraisals, condition may show direct associations with outcomes (including negative affect) that vary as a function of narcissistic admiration and narcissistic rivalry. We expected admiration to be associated with stronger differentiation between conditions on recognition-congruent outcomes, whereas rivalry was expected to be associated with stronger differentiation between conditions on threat and resistance outcomes.*

## 2. Materials and Methods

### 2.1. Participants

Participants were 874 young parents recruited from the general population in Israel via a well-established local online research panel (iPanel) and supplementary community-based recruitment channels. Eligibility criteria included age between 25 and 45 years (this age range was selected to capture parents who are likely to engage regularly in playground interactions while being developmentally embedded in early-to-mid parenthood), being married or cohabiting, and having at least one child within the age range typical for playground use (child age range = 1–12 years). Participants were required to report regular use of public playgrounds with their children and sufficient proficiency in Hebrew to complete the questionnaires and view video stimuli with audio enabled.

Participation was voluntary and data were collected through a secure online platform. After providing informed consent, participants completed a set of questionnaires, viewed the video stimulus, and then completed additional questionnaires. Participants were informed that they could discontinue participation at any time and that no personally identifying information would be collected. Compensation was provided in accordance with the panel’s standard practice (10 ILS).

Exclusion criteria were defined a priori and included univariate outliers (*n* = 17), highly inconsistent response patterns indexed by unusually large inter-item standard deviations (*n* = 47), and evidence of straightlining (i.e., providing the same response across a large number of items) identified via longstring analysis (*n* = 34). Participant exclusions and final cell sizes by condition are reported in full in Section 3. The final analytic sample consisted of 776 participants (49.9% women), randomly assigned to the recognition-based norm framing condition (*n* = 398) or the status-challenging norm framing condition (*n* = 378). Participants ranged in age from 25 to 41 years (*M* = 35.45, *SD* = 4.10). Descriptive statistics for education, employment status, income, religiosity, and family composition are reported in Table 1. No exclusions were made based on levels of narcissistic admiration or rivalry, which were treated as continuous individual-difference variables.

An a priori power analysis (G*Power 3.1) indicated that a minimum of 395 participants would be sufficient to detect small interaction effects (f^2^ = 0.02, α = 0.05, 1 − β = 0.80). The final sample size therefore provided adequate statistical power (>0.99) for all planned analyses, including tests of conditional indirect effects.

### 2.2. Materials

#### 2.2.1. Video Stimuli

Stimuli consisted of short, ultra-realistic cinematic videos depicting an everyday playground interaction. In each video, two child-like figures, one from each family, arrive at a playground slide at the same moment, creating an ambiguous but normatively common situation requiring regulation of a shared resource. To ensure compliance with platform constraints while preserving ecological plausibility, the child-like figures were presented as contextual background elements only and were never shown front-facing or in identifiable close-up; they did not speak in any version. These figures served solely as non-identifiable contextual cues to instantiate the turn-taking situation and were not visually emphasized (e.g., no close-ups, no frontal facial visibility, no unique identifiers). The scene was set in a public playground and designed to reflect naturalistic, everyday parenting interactions.

Two experimental conditions were implemented that differed only in the initiating parent’s norm framing (recognition-based vs. status-challenging). For production purposes, each condition was created in two speaker variants (father speaks; mother speaks). Speaker gender was not treated as an experimental factor; rather, it was used to match the initiating speaker’s gender to the participant’s gender at exposure. Across all video stimuli, only the initiating parent (Family A) both initiated the interaction and delivered the norm-framing statement. The other Family A parent remained present but silent, and Family B did not speak in any condition. This design ensured that the manipulation was confined to the framing content while holding all other contextual features constant.

In the recognition-based condition, the initiating parent framed the situation around fairness, shared standards, and mutual legitimacy (e.g., emphasizing turn-taking and concern that everyone feels okay). Authority was enacted through inclusive language, open posture, and affiliative tone. In the status-challenging condition, the initiating parent framed the same objective situation around priority claims and non-negotiability (e.g., asserting order and closing the possibility of discussion). Authority was enacted through a controlled, firm tone, restrained gesture use, sustained direct gaze toward the other parents, and subtle spatial positioning. Importantly, neither condition involved shouting, overt aggression, or emotional escalation.

The two conditions were designed to be normatively plausible and socially ordinary. The manipulation targeted the social meaning of norm enforcement (recognition-based versus status-challenging), rather than conflict intensity, rudeness, or emotional arousal.

#### 2.2.2. Final Stimulus Set and Standardization

The final stimulus set comprised four videos: two recognition-based versions and two status-challenging versions, each produced in two speaker variants (father speaks; mother speaks). Across all videos, the setting, slide, playground layout and vegetation, camera perspective, depth of field, lighting, ambient sound, pacing, and scene structure were constrained as tightly as technically possible. All versions used the same dialogue content within each framing condition, and the Hebrew subtitles matched the dialogue line-by-line. Dialogue content was held constant across speaker-gender variants within each condition, but it differed systematically between the recognition-based and status-challenging conditions to instantiate the intended norm-framing contrast. Within each norm-framing condition, the spoken dialogue and Hebrew subtitles were identical across speaker-gender variants; differences between variants were conveyed through delivery (tone, pacing, gaze, and spatial positioning), not through lexical content. To achieve the target duration while preserving equivalence, any additional dialogue used to extend pacing (e.g., brief reinforcement and closure lines) was added symmetrically across conditions and did not introduce new informational content beyond the intended framing.

Because AI video generation does not reliably permit frame-perfect replication across separate renders, the standardization goal was perceptual and narrative equivalence rather than literal frame identity. Accordingly, the videos were produced to appear as the same underlying scene unfolding in real time, with only the intended experimental difference varying: the initiating parent’s norm-framing dialogue and its associated delivery (tone, gaze behavior, and restrained micro-behavioral cues). Two speaker-gender variants were created solely for participant–stimulus matching and were not analyzed as a separate factor. Videos were matched on duration (approximately 50 s) and maintained an unhurried rhythm to allow comfortable reading of Hebrew subtitles.

**Figure 2:**
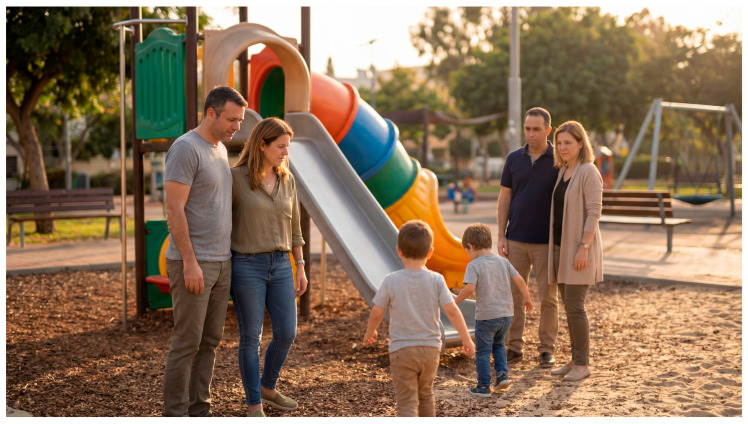
Illustrative stills from the standardized playground interaction, created by AI and used in the recognition-based and status-challenging norm-framing conditions. The full finalized videos and associated scripts are openly available in the OSF repository (https://osf.io/9uemc, accessed on 21 April 2026), where readers can access the stimulus materials for full contextual documentation. The visual materials were created specifically for this research, and the characters are synthetic and not intended to represent identifiable real individuals.

Participants were exposed only to finalized clips. No participants viewed multiple versions of the same scenario. Each participant viewed one full-screen clip only, ensuring that the manipulated variable was the norm framing enacted by the initiating parent. A concept illustration of the video-stimulus setting is provided in Figure 2.

#### 2.2.3. AI Video-Generation Tools and Production Pipeline

To create photorealistic stimuli while holding content constant, we used the same multi-platform AI video-generation pipeline employed in our prior video-based studies. Specifically, we used eight AI video platforms: Veo 3.1 (Google DeepMind, London, UK), Kling AI (Kuaishou Technology, Beijing, China), Sora 2 (OpenAI, San Francisco, CA, USA), Pictory (Bothell, WA, USA), InVideo AI (InVideo, San Francisco, CA, USA), Runway Gen-3 (Runway AI, New York City, NY, USA), HeyGen (HeyGen Technology Inc., Los Angeles, CA, USA), and Hailuo AI (MiniMax, Shanghai, China).

Multiple platforms were used because no single generator consistently produced high-quality outputs that simultaneously satisfied all constraints (stable characters, natural body movement, gaze behavior, lip-sync, and scene continuity) while preserving an identical script and environment. Using several tools allowed us to generate multiple candidate renderings of the same scripted interaction and then select the best-matched final clips for experimental use.

For each platform, we applied the same storyboard instructions, dialogue scripts, target duration, aspect ratio (16:9), and resolution (1080p), while constraining avatars’ appearance, camera framing, and background environment as tightly as technically possible. In addition, the production workflow prioritized within-scene reuse and variation generation from a stabilized base render when supported by a platform, to maximize continuity of the playground layout and character appearance across the four final videos. Following generation, final clips underwent identical post-processing procedures applied uniformly across conditions, including subtitle synchronization and quality control checks to confirm that Family B remained silent and that child-like figures were non-identifiable and non-speaking.

The use of multiple AI video-generation platforms served solely as a production strategy to obtain closely matched, ecologically valid stimuli under current technical constraints. All finalized clips were treated as standardized experimental stimuli rather than as objects of comparison, and no analyses were conducted with respect to platform-specific features or generation methods. Accordingly, the technological pipeline does not constitute a substantive variable in the study design, but rather a means of ensuring controlled and realistic stimulus presentation.

Although the study was not preregistered, materials relevant to reproducing the study are available in our OSF repository (https://osf.io/9uemc, accessed on 21 April 2026). The repository includes the finalized stimulus videos, the full dialogue scripts, storyboard prompts, production specifications, post-processing documentation, and the anonymized SPSS data file used for the reported analyses.

#### 2.2.4. Expert-Based Manipulation Check (Stimulus Validation)

To minimize demand characteristics and avoid conceptual overlap with the post-exposure outcomes, no participant-level manipulation-check items were administered. Instead, we validated the manipulation using independent expert ratings. An expert panel of eight judges (four men, four women) rated the finalized stimuli. Each judge viewed both videos (recognition-based vs. status-challenging). To preserve ecological naturalness and avoid introducing systematic variance due to speaker–judge gender mismatch, judge gender was matched to the initiating parent’s gender in each condition (male judges viewed the father-speaker versions; female judges viewed the mother-speaker versions). Judges were blind to the study hypotheses and rated each video on five 7-point dimensions: (a) the extent to which the clip conveyed status-challenging norms (entitlement, priority claims, implied hierarchy), (b) the extent to which it conveyed recognition-based norms (fairness, mutual legitimacy, respectful coordination), and (c) three general stimulus-quality dimensions: realism (how lifelike or credible the scene appeared), naturalness (how natural vs. scripted the dialogue sounded), and clarity (how clear the speaker’s message was).

Interrater agreement across judges was excellent for both focal norm-framing dimensions, as indicated by a two-way random-effects model with average measures (ICC [2,k]). Agreement was extremely high for both norm-framing dimensions (ICC = 1.00) and the recognition-based norms (ICC = 0.99; [29,30]), indicating highly consistent use of the rating scales across evaluators. As expected, the status-challenging video was rated substantially higher on status-challenging norms than the recognition-based video (*M* = 6.75, *SD* = 0.46 vs. *M* = 1.63, *SD* = 0.74), paired t(7) = 22.63, *p* < 0.001, *CI*_95%_[4.59, 5.66], Cohen’s dz = 8.00. Conversely, the recognition-based video was rated substantially higher on recognition-based norms than the status-challenging video (*M* = 6.63, *SD* = 0.52 vs. *M* = 2.50, *SD* = 0.76), paired t(7) = 18.20, *p* < 0.001, *CI*_95%_[3.59, 4.66], Cohen’s dz = 6.44.

The two videos did not differ significantly on the three general stimulus-quality dimensions: realism (status-challenging: *M* = 6.38, *SD* = 0.74; recognition-based: *M* = 6.75, *SD* = 0.71), paired t(7) = −1.43, *p* = 0.20, *CI*_95%_[−1.00, 0.25], Cohen’s dz = −0.50; clarity (status-challenging: *M* = 6.88, *SD* = 0.35; recognition-based: *M* = 6.75, *SD* = 0.46), paired t(7) = 1.00, *p* = 0.35, *CI*_95%_[−0.17, 0.42], Cohen’s dz = 0.35; and naturalness (status-challenging: *M* = 6.00, *SD* = 0.53; recognition-based: *M* = 6.00, *SD* = 0.76), paired t(7) = 0.00, *p* = 1.00, *CI*_95%_[−0.77, 0.77], Cohen’s dz = 0.00.

Together, these expert ratings confirm that the two stimuli differed strongly and selectively on the intended norm-framing dimension (recognition-based vs. status-challenging), while being comparable in realism, naturalness, and clarity. This pattern supports the interpretation that the manipulation affected norm framing rather than reflecting general differences in production quality or comprehensibility.

#### 2.2.5. Pre-Manipulation Questionnaires

##### Demographic Questionnaire

Participants completed a demographic questionnaire assessing age, gender, education level, employment status, household income, religiosity, marital status, and number of children. Given the playground context, participants also reported background variables relevant to exposure to playground interactions, including frequency of playground use and the age of a focal child. Specifically, participants reported how often they take their child(ren) to a playground using a single item rated on a 7-point scale ranging from 1 (very little) to 7 (very much). Participants were then instructed to think about one specific child when considering the playground scenario, and they reported the focal child’s age as an open-ended response (years). For participants with multiple children, the focal child was defined as the child most relevant to playground use.

##### Narcissistic Admiration and Rivalry Questionnaire

In addition, in the pre-manipulation stage, participants completed the Narcissistic Admiration and Rivalry Questionnaire (NARQ; [1]). The NARQ consists of two subscales, narcissistic admiration (9 items) and narcissistic rivalry (9 items). Items were rated on a 6-point Likert scale ranging from 1 (not agree at all) to 6 (agree completely). Subscale scores were computed as the mean of the relevant items, with higher scores indicating greater levels of admiration or rivalry. Internal consistency in the present study was α = 0.82 for narcissistic admiration and α = 0.83 for narcissistic rivalry.

All questionnaires were administered in Hebrew. Measures originally developed in English and did not have established Hebrew versions were translated using a standard translation–back-translation procedure to ensure semantic and conceptual equivalence.

#### 2.2.6. Post-Manipulation Questionnaires

##### Perspective-Taking Instructions (Post-Video Measures)

Immediately after video exposure, participants received standardized perspective-taking instructions. Specifically, they were asked to respond to all post-video measures as if they were the parent from the family that did not respond verbally in the clip (Family B), facing the parents who initiated the interaction. Participants were further instructed to base their responses on their own feelings and reactions as a parent in the depicted situation, rather than offering a third-person evaluation of the other parents or the situation. These instructions were used to standardize the appraisal perspective across respondents. Participants then completed a set of measures assessing appraisals and immediate responses to the situation.

##### Perceived Recognition

Perceived recognition was assessed using the Perceived Recognition Scale (PRS; [20,21]). The scale includes six items capturing the extent to which participants felt seen, acknowledged, respected, and treated as legitimate by the initiating parent in the video. For the present study, items were contextualized to the playground scenario by referring consistently to the parent who initiated the interaction in the video. Responses were provided on a 7-point scale (1 = very little, 7 = very much). Items were averaged to compute a composite PRS score, with higher values indicating greater perceived recognition. Internal consistency in the current sample was α = 0.96.

##### Perceived Freedom Threat

Perceived freedom threat (PFT) was assessed using four items adapted from Dillard and Shen (2005) [22]. Items captured the extent to which the message conveyed by the parent who initiated the interaction was experienced as restricting freedom, imposing constraints, or exerting coercive influence. Responses were recorded on a 5-point Likert scale (1 = not at all, 5 = very much). Items were averaged to create a composite perceived freedom threat score, with higher values indicating greater perceived threat. Internal consistency in the current sample was α = 0.81.

##### State Reactance

State reactance was assessed using a contextualized version of the Salzburger State Reactance Scale (SSR; [25]). The scale comprises 10 items capturing immediate emotional reactance, resistance tendencies, and negative evaluations directed toward the source of the message. For the present study, items were contextualized to refer to the parent who initiated the interaction in the playground scenario, and participants responded from the perspective of the focal parent (i.e., a parent from the family being spoken to in the interaction; Family B). Responses were recorded on a 5-point Likert scale (1 = not at all, 5 = very much). Items were averaged to compute a composite state reactance score, with higher values indicating greater reactance. Internal consistency in the current sample was α = 0.97.

##### Negative Affect

Immediate affect was assessed using a brief set of contextualized items anchored in the positive and negative affect framework [31]. This brief index was inspired by that framework rather than intended as a full administration of the PANAS instrument. It was designed to capture immediate, context-specific affective reactions to the playground scenario while minimizing participant burden and maintaining temporal proximity to the stimulus. Higher scores reflected more negative affect. The item set consisted of three negative affect items (i.e., anger, irritation/discomfort, frustration) and two positive affect items (i.e., calmness and feeling at ease), all rated from the perspective of the focal parent (Family B). Responses were recorded on a 5-point Likert scale (1 = not at all, 5 = very much). Positive affect items were reverse-coded, and all items were averaged to compute a single negative affect index, with higher values indicating more negative affective responding to the scenario. Internal consistency in the current sample was α = 0.91.

##### Evaluation of the Initiating Parent

Participants evaluated the behavior of the parent who initiated the interaction using a contextualized semantic differential format [32]. Specifically, participants rated the initiating parent on five bipolar adjective pairs (fair–unfair, considerate–inconsiderate, respectful–disrespectful, legitimate–illegitimate, appropriate–inappropriate) using 7-point scales. Ratings were completed from the perspective of the focal parent (Family B). Items were scored such that higher values indicated more positive evaluations and were averaged to form a composite evaluation score. Internal consistency in the current sample was α = 0.97.

##### Behavioral Preferences

Behavioral preferences were assessed using six scenario-specific, theory-driven items designed to capture two distinct response tendencies in the playground context: recognition-based preferences and entitlement-based preferences. Items were contextualized to the clip and asked participants, from the perspective of the focal parent (Family B), how they would prefer to respond in the situation. Responses were recorded on a 5-point Likert scale (1 = not at all, 5 = very much). Items were organized into two indices, recognition-based preferences (three items emphasizing fairness, turn-taking, and mutual agreement) and entitlement-based preferences (three items emphasizing priority claims or unilateral control). This conceptual distinction is grounded in reactance theory, which links perceived restrictions of freedom to motivations to restore autonomy through oppositional or controlling responses, e.g., [22], and in process models of narcissistic self-regulation and status pursuit that differentiate between recognition-oriented versus defensive status-protective responding [1,2]. Items were pretested for clarity and face validity. Items were averaged to create behavioral preference indices (α = 0.89 for recognition-based preferences; α = 0.74 for entitlement-based preferences).

Conceptually, these behavioral preference indices were intended to capture action tendencies rather than affective states. Whereas state reactance and negative affect reflect emotional and cognitive resistance, recognition-based and entitlement-based preferences represent alternative regulatory strategies for managing shared resources in public contexts. Recognition-based preferences emphasize coordination and mutual legitimacy, whereas entitlement-based preferences emphasize unilateral control or priority assertion. This distinction aligns with process models of narcissistic self-regulation, which differentiate between affiliative status seeking and defensive, status-protective responding [1,2]. Thus, the behavioral indices were designed to extend beyond affect and evaluation by indexing motivational direction in the immediate situation.

### 2.3. Procedure

After providing informed consent, participants completed the demographic questionnaire and the NARQ. They were then randomly assigned to one of the two norm-framing conditions and viewed the video stimulus. Immediately after viewing the clip, participants completed the appraisal and response measures in a fixed order to preserve temporal proximity to the stimulus and minimize retrospection. At the end of the study, participants were debriefed and thanked for their participation.

### 2.4. Analytic Strategy

Analyses proceeded in several stages. We began by computing descriptive statistics and zero-order Pearson correlations among all study variables. Given that all focal constructs were measured via self-report within a single assessment session, we examined the potential for common method variance (CMV). Specifically, we conducted Harman’s single-factor test by entering all appraisal and response items into an unrotated exploratory factor analysis. We then conducted a series of conditional process (i.e., moderated mediation) analyses to test our primary hypotheses. Specifically, we expected narcissistic admiration and narcissistic rivalry to moderate the associations between experimental condition (predictor) and the proposed mediators, perceived recognition and perceived freedom threat, which, in turn, were expected to predict the outcome variables. These interaction tests correspond to H5a, whereas the tests of conditional direct effects on the outcomes correspond to H5b. The analytic materials corresponding to these procedures are available in the OSF repository and include the anonymized SPSS data file used for the reported analyses. These models were estimated using PROCESS Model 10 [33] in SPSS (Version 31). Experimental condition was effect-coded (recognition-based = −1; status-challenging = +1). Narcissistic admiration and narcissistic rivalry were entered as continuous moderators. All continuous predictors were mean centered prior to analysis.

Separate models were estimated for each outcome variable because the outcomes were theory-derived and intended to capture conceptually distinct immediate response domains rather than interchangeable indicators of a single latent construct. Specifically, state reactance and negative affect indexed affective resistance, evaluation of the initiating parent indexed source appraisal, and the behavioral preference measures indexed motivational direction in the immediate situation. Although several outcomes were moderately to strongly intercorrelated, this overlap was not uniform across all variables, and the behavioral preference indices had weaker associations with the affective-evaluative outcomes and did not show identical experimental condition effects. Accordingly, we retained separate outcome models in order to preserve theoretically meaningful distinctions that could have been obscured by data reduction. The outcomes were state reactance, negative affect, evaluation of the initiating parent, recognition-based preferences, and entitlement-based preferences.

Conditional indirect effects were evaluated using bias-corrected bootstrapped confidence intervals based on 10,000 resamples. For each pathway, we report (a) regression coefficients and focal interaction terms, (b) bootstrapped indirect effects, and (c) the index of moderated mediation along with conditional indirect effects within each experimental condition. Model *R*^2^ and omnibus *F* statistics are provided in the corresponding tables. Significant interactions were probed using simple slopes analyses at ±1 *SD* of the moderator. Effect sizes and confidence intervals are reported alongside *p* values.

### 2.5. Ethics Statement

The study adhered to the ethical principles for research involving human participants, consistent with the Declaration of Helsinki and subsequent amendments. Ethical approval was obtained from the Institutional Review Board (IRB) of the Jerusalem Multidisciplinary Academic Center (approval no. 715; 26 January 2026). All participants provided informed consent prior to taking part in the study. Participation was voluntary, data were collected anonymously, and participants were free to discontinue participation at any point without any consequences.

## 3. Results

### 3.1. Background and Sociodemographic Variables

Table 1 presents the sociodemographic and background characteristics of the sample for the full sample and stratified by gender and experimental condition. Reported variables include participant age, number of children, age of the focal child, educational attainment, employment status, marital status, household income, and religiosity. These data are provided to characterize the sample and to contextualize the experimental findings.

To evaluate the equivalence of the experimental groups, we conducted independent-samples *t* tests for continuous variables and χ^2^ tests for categorical variables. No statistically significant differences emerged between the recognition-based and status-challenging conditions on any sociodemographic or background characteristic (all *p* > 0.05).

This pattern supports the internal validity of the experimental comparison by reducing the likelihood that any observed condition effects were attributable to pre-existing group differences. Accordingly, these variables were not included as covariates in the primary analyses.

### 3.2. Univariate Analyses

Descriptive statistics are reported in Table 2A, and zero-order correlations are reported in Table 2B. Together, these tables provide an overview of the distributions of the study variables within each condition and of their bivariate associations across conditions.

The descriptive statistics indicated no marked departures from normality across conditions, supporting the use of the planned parametric analyses. Although several appraisal and affective-evaluative variables were strongly intercorrelated, this pattern was not uniform across all of the outcome variables. In particular, the behavioral preference indices showed comparatively weaker associations with the affective-evaluative variables, and the two behavioral preference indices were not strongly related to one another across conditions. This pattern supports treating the outcomes as related but conceptually distinguishable indicators of immediate response rather than collapsing them into a smaller set of aggregate factors.

Narcissistic admiration exhibited small-to-moderate positive correlations with narcissistic rivalry and entitlement-based preferences in both experimental conditions. Additionally, narcissistic admiration was positively associated with recognition-based preferences in the status-challenging condition, but it was not significantly related to recognition-based preferences in the recognition-based condition.

Narcissistic rivalry showed small-to-moderate positive correlations with state reactance and entitlement-based preferences across both conditions. In contrast, narcissistic rivalry had small-to-moderate negative correlations with recognition-based preferences in both experimental conditions.

To assess potential common method variance (CMV), we conducted Harman’s single-factor test by entering all measurement items into an unrotated principal component analysis. The first unrotated factor accounted for 36.03% of the total variance, which is well below the conventional 50% threshold typically interpreted as indicating substantial common method bias. This result suggests that a single general factor does not dominate the covariance structure of the data. Accordingly, common method variance is unlikely to represent a serious threat to the validity of the observed associations.

As shown in Table 3, participants in the recognition-based and status-challenging conditions did not differ in levels of narcissistic admiration or narcissistic rivalry. This pattern suggests that random assignment was successful in producing groups that were comparable on these dispositional traits.

Consistent with expectations, participants in the recognition-based condition reported significantly greater perceived recognition than those in the status-challenging condition, indicating that the manipulation functioned as intended. They also evaluated the initiating parent more favorably.

In contrast, participants in the status-challenging condition reported higher levels of perceived freedom threat, state reactance, negative affect, and entitlement-based preferences relative to those in the recognition-based condition. The two conditions did not differ with respect to recognition-based preferences.

**Table 3:** Comparisons of the recognition-based and status-challenging conditions.

	Recognition-Based Condition (*n* = 398)		Status-Challenging Condition (*n* = 378)			
	*M*	*SD*	*M*	*SD*	*t*_(774)_	*p*
Narcissistic Admiration	3.49	0.79	3.50	0.83	−0.17	0.865
Narcissistic Rivalry	2.07	0.75	2.08	0.76	−0.27	0.787
Perceived Recognition	5.10	1.47	3.01	1.43	20.12	<0.001
Perceived Freedom Threat	2.84	0.99	3.57	0.93	−10.61	<0.001
State Reactance	2.12	1.04	3.42	0.99	−17.80	<0.001
Negative Affect	2.41	1.01	3.64	0.89	−18.03	<0.001
Evaluation of the Initiating Parent	5.11	1.66	2.81	1.54	19.92	<0.001
Recognition-Based Preferences	4.28	0.81	4.16	0.84	1.88	0.060
Entitlement-Based Preferences	2.74	0.95	2.96	0.99	−3.21	0.001

Note. Group differences were tested using two-tailed independent-samples *t* tests.

### 3.3. Perceived Recognition and Perceived Freedom Threat

The conditional process analyses indicated that experimental condition was significantly associated with both proposed mediators. Specifically, condition was negatively related to perceived recognition (*B* = −0.59, *CI*_95%_[−0.64, −0.53], *SE* = 0.03, *t* = −20.15, *p* < 0.001) and positively related to perceived freedom threat (*B* = 0.36, *CI*_95%_[0.29, 0.42], *SE* = 0.03, *t* = 10.60, *p* < 0.001). Participants in the status-challenging condition therefore reported lower perceived recognition and greater perceived freedom threat than those in the recognition-based condition.

In contrast, narcissistic admiration was not associated with perceived recognition (*B* = 0.03, *CI*_95%_[−0.02, 0.09], *SE* = 0.03, *t* = 1.17, *p* = 0.241) or perceived freedom threat (*B* = 0.04, *CI*_95%_[−0.03, 0.11], *SE* = 0.03, *t* = 1.12, *p* = 0.265). Moreover, narcissistic admiration did not moderate the association between experimental condition and perceived recognition (*B* = −0.02, *CI*_95%_[−0.08, 0.03], *SE* = 0.03, *t* = −0.80, *p* = 0.427) or perceived freedom threat (*B* = 0.03, *CI*_95%_[−0.03, 0.10], *SE* = 0.03, *t* = 1.01, *p* = 0.312).

Similarly, narcissistic rivalry was not associated with perceived recognition (*B* = 0.02, *CI*_95%_[−0.03, 0.08], *SE* = 0.03, *t* = 0.83, *p* = 0.406) or perceived freedom threat (*B* = 0.06, *CI*_95%_[−0.01, 0.12], *SE* = 0.03, *t* = 1.69, *p* = 0.092). Narcissistic rivalry also did not moderate the effects of experimental condition on perceived recognition (*B* = 0.04, *CI*_95%_[−0.01, 0.10], *SE* = 0.03, *t* = 1.52, *p* = 0.130) or perceived freedom threat (*B* = −0.01, *CI*_95%_[−0.08, 0.06], *SE* = 0.03, *t* = −0.25, *p* = 0.805).

Notably, neither narcissistic admiration nor narcissistic rivalry moderated the effects of condition on the appraisal layer. Within the scope of the grandiose narcissism dimensions assessed here, this pattern suggests that recognition-based versus status-challenging norm framing exerted broadly similar situational effects on immediate appraisals. However, this should not be taken to mean that personality is irrelevant to these processes more generally. Rather, the present findings indicate that the appraisal effects of the manipulation were not contingent on variation in admiration and rivalry as measured in this study.

In the following sections, Table 4, Table 5, Table 6, Table 7 and Table 8 follow a parallel reporting structure to facilitate comparison across models. Each table presents: (a) the mediator model predicting perceived recognition, (b) the mediator model predicting perceived freedom threat, and (c) the outcome model predicting one focal dependent variable (i.e., state reactance, negative affect, evaluation of the initiating parent, recognition-based preferences, or entitlement-based preferences). Because the mediator models are identical across analyses, they are reproduced in each table for completeness, including *R*^2^ values and omnibus *F* tests. The outcome models differ only with respect to the dependent variable. Accordingly, the text emphasizes hypothesis-relevant effects, including interaction terms (when applicable), bootstrapped indirect effects with 95% confidence intervals, and the index of moderated mediation.

### 3.4. State Reactance

The results of the conditional process analysis predicting state reactance are presented in Table 4. Experimental condition was positively associated with state reactance (*B* = 0.13, *CI*_95%_[0.08, 0.17], *SE* = 0.02, *t* = 5.27, *p* < 0.001), such that participants in the status-challenging condition reported higher levels of state reactance than those in the recognition-based condition. Both narcissistic admiration (*B* = 0.06, *CI*_95%_[0.02, 0.09], *SE* = 0.02, *t* = 2.82, *p* = 0.005) and narcissistic rivalry (*B* = 0.10, *CI*_95%_[0.06, 0.14], *SE* = 0.02, *t* = 4.95, *p* < 0.001) were also positively associated with state reactance. However, neither narcissistic admiration (*B* = 0.00, *CI*_95%_[−0.04, 0.04], *SE* = 0.02, *t* = −0.10, *p* = 0.919) nor narcissistic rivalry (*B* = 0.00, *CI*_95%_[−0.04, 0.04], *SE* = 0.02, *t* = 0.02, *p* = 0.986) moderated the effect of experimental condition on state reactance. With respect to the mediators, perceived recognition was negatively associated with state reactance (*B* = −0.47, *CI*_95%_[−0.53, −0.42], *SE* = 0.03, *t* = −17.02, *p* < 0.001), whereas perceived freedom threat was positively associated with state reactance (*B* = 0.38, *CI*_95%_[0.33, 0.42], *SE* = 0.02, *t* = 15.68, *p* < 0.001).

Experimental condition also demonstrated significant indirect associations with state reactance through both mediators. Specifically, condition had a positive indirect effect via perceived recognition (*B* = 0.27, *CI*_95%_[0.22, 0.31], *SE* = 0.02, *z* = 12.58, *p* < 0.001). However, this indirect association was not moderated by narcissistic admiration (*B* = 0.01, *CI*_95%_[−0.02, 0.04], *SE* = 0.02) or narcissistic rivalry (*B* = −0.02, *CI*_95%_[−0.05, 0.00], *SE* = 0.01).

Similarly, experimental condition had a positive indirect association with state reactance through perceived freedom threat (*B* = 0.14, *CI*_95%_[0.11, 0.17], *SE* = 0.02, *z* = 8.87, *p* < 0.001). Again, this indirect association was not moderated by narcissistic admiration (*B* = 0.01, *CI*_95%_[−0.01, 0.04], *SE* = 0.01) or narcissistic rivalry (*B* = 0.00, *CI*_95%_[−0.03, 0.02], *SE* = 0.01).

**Table 4:** Results of the conditional process analysis for state reactance.

	Outcome								
	M_1_: Perceived Recognition			M_2_: Perceived Freedom Threat			Y: State Reactance		
Predictor	*B*	*SE*	*p*	*B*	*SE*	*p*	*B*	*SE*	*p*
X: Experimental Condition	−0.59	0.03	<0.001	0.36	0.03	<0.001	0.13	0.02	<0.001
W_1_: Narcissistic Admiration (ADM)	0.03	0.03	0.241	0.04	0.03	0.265	0.06	0.02	0.005
W_2_: Narcissistic Rivalry (RIV)	0.02	0.03	0.406	0.06	0.03	0.092	0.10	0.02	<0.001
M_1_: Perceived Recognition	–	–	–	–	–	–	−0.47	0.03	<0.001
M_2_: Perceived Freedom Threat	–	–	–	–	–	–	0.38	0.02	<0.001
X × W_1_: Condition × ADM	−0.02	0.03	0.427	0.03	0.03	0.265	0.00	0.02	0.919
X × W_2_: Condition × RIV	0.02	0.03	0.406	−0.01	0.03	0.805	0.00	0.02	0.986
Constant	−0.02	0.03	0.597	0.01	0.03	0.788	0.00	0.02	0.866
	*R*^2^ = 0.35			*R*^2^ = 0.13			*R*^2^ = 0.71		
	*F* = 82.06, *p* < 0.001			*F* = 23.76, *p* < 0.001			*F* = 271.30, *p* < 0.001		
Conditional Indirect Association of Experimental Condition with State Reactance through Perceived Recognition									
Condition	*Coeff.*		*Boot SE*		*Boot LCI*		*Boot UCI*		
Low Narcissistic Admiration	0.27		0.03		0.22		0.32		
High Narcissistic Admiration	0.29		0.03		0.24		0.35		
Low Narcissistic Rivalry	0.30		0.03		0.24		0.36		
High Narcissistic Rivalry	0.25		0.03		0.21		0.31		
Conditional Indirect Association of Experimental Condition with State Reactance through Perceived Freedom Threat									
Condition	*Coeff.*		*Boot SE*		*Boot LCI*		*Boot UCI*		
Low Narcissistic Admiration	0.12		0.02		0.08		0.16		
High Narcissistic Admiration	0.15		0.02		0.11		0.19		
Low Narcissistic Rivalry	0.14		0.02		0.10		0.18		
High Narcissistic Rivalry	0.13		0.02		0.10		0.17		

### 3.5. Negative Affect

The results of the conditional process analysis predicting negative affect are presented in Table 5. Experimental condition was positively associated with negative affect (*B* = 0.14, *CI*_95%_[0.09, 0.19], *SE* = 0.03, *t* = 5.33, *p* < 0.001), such that participants in the status-challenging condition reported higher levels of negative affect than those in the recognition-based condition. Narcissistic rivalry was also positively associated with negative affect (*B* = 0.05, *CI*_95%_[0.01, 0.09], *SE* = 0.02, *t* = 2.38, *p* = 0.018), but narcissistic admiration was not (*B* = 0.00, *CI*_95%_[−0.04, 0.05], *SE* = 0.02, *t* = 0.14, *p* = 0.888). However, neither narcissistic admiration (*B* = 0.01, *CI*_95%_[−0.04, 0.05], *SE* = 0.02, *t* = 0.31, *p* = 0.759) nor narcissistic rivalry (*B* = −0.01, *CI*_95%_[−0.05, 0.03], *SE* = 0.02, *t* = −0.39, *p* = 0.695) moderated the effect of experimental condition on negative affect. With respect to the mediators, perceived recognition was negatively associated with negative affect (*B* = −0.51, *CI*_95%_[−0.57, −0.45], *SE* = 0.03, *t* = −16.95, *p* < 0.001), whereas perceived freedom threat was positively associated with negative affect (*B* = 0.30, *CI*_95%_[0.25, 0.35], *SE* = 0.03, *t* = 11.40, *p* < 0.001).

Experimental condition also demonstrated significant indirect associations with negative affect through both mediators. Specifically, condition had a positive indirect effect via perceived recognition (*B* = 0.30, *CI*_95%_[0.25, 0.34], *SE* = 0.02, *z* = 12.92, *p* < 0.001). However, this indirect association was not moderated by narcissistic admiration (*B* = 0.01, *CI*_95%_[−0.02, 0.04], *SE* = 0.02) or narcissistic rivalry (*B* = −0.02, *CI*_95%_[−0.05, 0.01], *SE* = 0.01).

Similarly, experimental condition had a positive indirect association with negative affect through perceived freedom threat (*B* = 0.11, *CI*_95%_[0.08, 0.14], *SE* = 0.01, *z* = 7.85, *p* < 0.001). Again, this indirect association was not moderated by narcissistic admiration (*B* = 0.01, *CI*_95%_[−0.01, 0.03], *SE* = 0.01) or narcissistic rivalry (*B* = 0.00, *CI*_95%_[−0.02, 0.02], *SE* = 0.01).

**Table 5:** Results of the conditional process analysis for negative affect.

	Outcome								
	M_1_: Perceived Recognition			M_2_: Perceived Freedom Threat			Y: Negative Affect		
Predictor	*B*	*SE*	*p*	*B*	*SE*	*p*	*B*	*SE*	*p*
X: Experimental Condition	−0.59	0.03	<0.001	0.36	0.03	<0.001	0.14	0.03	<0.001
W_1_: Narcissistic Admiration (ADM)	0.03	0.03	0.241	0.04	0.03	0.265	0.00	0.02	0.888
W_2_: Narcissistic Rivalry (RIV)	0.02	0.03	0.406	0.06	0.03	0.092	0.05	0.02	0.018
M_1_: Perceived Recognition	–	–	–	–	–	–	−0.51	0.03	<0.001
M_2_: Perceived Freedom Threat	–	–	–	–	–	–	0.30	0.03	<0.001
X × W_1_: Condition × ADM	−0.02	0.03	0.427	0.03	0.03	0.265	0.00	0.02	0.759
X × W_2_: Condition × RIV	0.02	0.03	0.406	−0.01	0.03	0.805	−0.01	0.02	0.695
Constant	−0.02	0.03	0.597	0.01	0.03	0.788	0.00	0.02	0.864
	*R*^2^ = 0.35			*R*^2^ = 0.13			*R*^2^ = 0.66		
	*F* = 82.06, *p* < 0.001			*F* = 23.76, *p* < 0.001			*F* = 214.54, *p* < 0.001		
Conditional Indirect Association of Experimental Condition with Negative Affect through Perceived Recognition									
Condition	*Coeff.*		*Boot SE*		*Boot LCI*		*Boot UCI*		
Low Narcissistic Admiration	0.29		0.03		0.24		0.35		
High Narcissistic Admiration	0.31		0.03		0.26		0.38		
Low Narcissistic Rivalry	0.32		0.03		0.26		0.38		
High Narcissistic Rivalry	0.27		0.03		0.22		0.33		
Conditional Indirect Association of Experimental Condition with Negative Affect through Perceived Freedom Threat									
Condition	*Coeff.*		*Boot SE*		*Boot LCI*		*Boot UCI*		
Low Narcissistic Admiration	0.10		0.02		0.06		0.13		
High Narcissistic Admiration	0.12		0.02		0.08		0.16		
Low Narcissistic Rivalry	0.11		0.02		0.07		0.15		
High Narcissistic Rivalry	0.10		0.02		0.07		0.14		

### 3.6. Evaluation of the Initiating Parent

**Figure 3:**
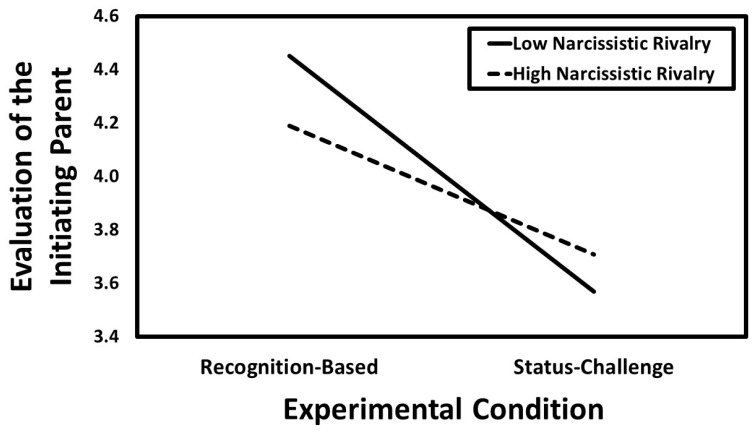
Predicted values illustrating the interaction that experimental condition had with narcissistic rivalry for evaluation of the initiating parent.

The results of the conditional process analysis predicting evaluation of the initiating parent are presented in Table 6. Experimental condition was negatively associated with evaluation of the initiating parent (*B* = −0.17, *CI*_95%_[−0.22, −0.12], *SE* = 0.03, *t* = −6.76, *p* < 0.001), such that participants in the status-challenging condition reported less positive evaluations of the initiating parent than those in the recognition-based condition. Neither narcissistic admiration (*B* = −0.04, *CI*_95%_[−0.08, 0.00], *SE* = 0.02, *t* = −1.77, *p* = 0.077) nor narcissistic rivalry (*B* = −0.02, *CI*_95%_[−0.06, 0.03], *SE* = 0.02, *t* = −0.73, *p* = 0.465) were associated with evaluation of the initiating parent. Although narcissistic admiration did not moderate the effect of experimental condition on evaluation of the initiating parent (*B* = −0.03, *CI*_95%_[−0.07, 0.01], *SE* = 0.02, *t* = −1.62, *p* = 0.105), narcissistic rivalry did moderate this association (*B* = 0.05, *CI*_95%_[0.01, 0.09], *SE* = 0.02, *t* = 2.44, *p* = 0.015; see Figure 3). Simple slopes analyses indicated that experimental condition was negatively associated with evaluation of the initiating parent when the level of narcissistic rivalry was high (*B* = −0.12, *CI*_95%_[−0.18, −0.06], *SE* = 0.03, *t* = −3.72, *p* < 0.001) but this association was even stronger when the level of narcissistic rivalry was low (*B* = −0.22, *CI*_95%_[−0.29, −0.16], *SE* = 0.03, *t* = −6.67, *p* < 0.001).

With respect to the mediators, perceived recognition was positively associated with evaluation of the initiating parent (*B* = 0.61, *CI*_95%_[0.56, 0.67], *SE* = 0.03, *t* = 20.87, *p* < 0.001). In contrast, perceived freedom threat was negatively associated with evaluation of the initiating parent (*B* = −0.14, *CI*_95%_[−0.19, −0.09], *SE* = 0.03, *t* = −5.51, *p* < 0.001).

Experimental condition also demonstrated significant indirect associations with evaluation of the initiating parent through both mediators. Specifically, condition had a negative indirect effect via perceived recognition (*B* = −0.36, *CI*_95%_[−0.41, −0.31], *SE* = 0.03, *z* = −14.47, *p* < 0.001). However, this indirect association was not moderated by narcissistic admiration (*B* = −0.01, *CI*_95%_[−0.05, 0.02], *SE* = 0.02) or narcissistic rivalry (*B* = 0.03, *CI*_95%_[−0.01, 0.06], *SE* = 0.02).

Similarly, experimental condition had a positive indirect association with evaluation of the initiating parent through perceived freedom threat (*B* = −0.05, *CI*_95%_[−0.08, −0.03], *SE* = 0.01, *z* = −5.04, *p* < 0.001). Again, this indirect association was not moderated by narcissistic admiration (*B* = 0.00, *CI*_95%_[−0.02, 0.01], *SE* = 0.01) or narcissistic rivalry (*B* = 0.00, *CI*_95%_[−0.01, 0.01], *SE* = 0.01).

**Table 6:** Results of the conditional process analysis for evaluation of the initiating parent.

	Outcome									
	M_1_: Perceived Recognition			M_2_: Perceived Freedom Threat			Y: Evaluation of the Initiating Parent			
Predictor	*B*	*SE*	*p*	*B*	*SE*	*p*	*B*		*SE*	*p*
X: Experimental Condition	−0.59	0.03	<0.001	0.36	0.03	<0.001	−0.17		0.03	<0.001
W_1_: Narcissistic Admiration (ADM)	0.03	0.03	0.241	0.04	0.03	0.265	−0.04		0.02	0.077
W_2_: Narcissistic Rivalry (RIV)	0.02	0.03	0.406	0.06	0.03	0.092	−0.02		0.02	0.465
M_1_: Perceived Recognition	–	–	–	–	–	–	0.61		0.03	<0.001
M_2_: Perceived Freedom Threat	–	–	–	–	–	–	−0.14		0.03	<0.001
X × W_1_: Condition × ADM	−0.02	0.03	0.0427	0.03	0.03	0.265	−0.03		0.02	0.105
X × W_2_: Condition × RIV	0.02	0.03	0.406	−0.01	0.03	0.805	0.05		0.02	0.015
Constant	−0.02	0.03	0.597	0.01	0.03	0.788	0.00		0.02	0.818
	*R*^2^ = 0.35			*R*^2^ = 0.13			*R*^2^ = 0.68			
	*F* = 82.06, *p* < 0.001			*F* = 23.76, *p* < 0.001			*F* = 229.00, *p* < 0.001			
Conditional Indirect Association of Experimental Condition with Evaluation of the Initiating Parent through Perceived Recognition										
Condition	*Coeff.*		*Boot SE*		*Boot LCI*			*Boot UCI*		
Low Narcissistic Admiration	−0.35		0.03		−0.41			−0.29		
High Narcissistic Admiration	−0.38		0.03		−0.45			−0.31		
Low Narcissistic Rivalry	−0.39		0.03		−0.45			−0.32		
High Narcissistic Rivalry	−0.33		0.03		−0.39			−0.27		
Conditional Indirect Association of Experimental Condition with Evaluation of the Initiating Parent through Perceived Freedom Threat										
Condition	*Coeff.*		*Boot SE*		*Boot LCI*			*Boot UCI*		
Low Narcissistic Admiration	−0.05		0.01		−0.07			−0.02		
High Narcissistic Admiration	−0.06		0.01		−0.08			−0.03		
Low Narcissistic Rivalry	−0.05		0.01		−0.08			−0.03		
High Narcissistic Rivalry	−0.05		0.01		−0.07			−0.03		

### 3.7. Recognition-Based Preferences

The results of the conditional process analysis predicting recognition-based preferences are presented in Table 7. Experimental condition was not significantly associated with recognition-based preferences (*B* = 0.03, *CI*_95%_[−0.06, 0.11], *SE* = 0.04, *t* = 0.63, *p* = 0.528). Narcissistic admiration was positively associated with recognition-based preferences (*B* = 0.11, *CI*_95%_[0.04, 0.18], *SE* = 0.04, *t* = 3.15, *p* = 0.002), whereas narcissistic rivalry was negatively associated with recognition-based preferences (*B* = −0.19, *CI*_95%_[−0.26, 0.13], *SE* = 0.04, *t* = −5.56, *p* < 0.001). However, neither narcissistic admiration (*B* = 0.02, *CI*_95%_[−0.05, 0.09], *SE* = 0.03, *t* = 0.51, *p* = 0.608) nor narcissistic rivalry (*B* = −0.07, *CI*_95%_[−0.13, 0.00], *SE* = 0.04, *t* = −1.87, *p* = 0.063) moderated the effect of experimental condition on recognition-based preferences. With respect to the mediators, perceived recognition (*B* = 0.29, *CI*_95%_[0.19, 0.39], *SE* = 0.05, *t* = 5.86, *p* < 0.001) and perceived freedom threat (*B* = 0.21, *CI*_95%_[0.13, 0.30], *SE* = 0.04, *t* = 4.99, *p* < 0.001) were positively associated with recognition-based preferences.

Experimental condition had significant indirect associations with recognition-based preferences through both mediators. Specifically, condition had a negative indirect effect via perceived recognition (*B* = −0.16, *CI*_95%_[−0.22, −0.10], *SE* = 0.03, *z* = −5.27, *p* < 0.001). However, this indirect association was not moderated by narcissistic admiration (*B* = −0.01, *CI*_95%_[−0.03, 0.01], *SE* = 0.01) or narcissistic rivalry (*B* = 0.01, *CI*_95%_[0.00, 0.03], *SE* = 0.01).

Experimental condition also had a positive indirect association with recognition-based preferences through perceived freedom threat (*B* = 0.07, *CI*_95%_[0.04, 0.11], *SE* = 0.02, *z* = 4.21, *p* < 0.001). Again, this indirect association was not moderated by narcissistic admiration (*B* = 0.01, *CI*_95%_[−0.01, 0.02], *SE* = 0.01) or narcissistic rivalry (*B* = 0.00, *CI*_95%_[−0.02, 0.01], *SE* = 0.01). Notably, although higher perceived recognition was associated with stronger recognition-based preferences as expected, perceived freedom threat was also positively associated with these preferences. This pattern suggests that recognition-based response tendencies may, in some cases, reflect attempts to restore coordination or legitimacy under perceived interpersonal pressure rather than simply the absence of autonomy threat.

**Table 7:** Results of the conditional process analysis for recognition-based preferences.

	Outcome								
	M_1_: Perceived Recognition			M_2_: Perceived Freedom Threat			Y: Recognition-Based Preferences		
Predictor	*B*	*SE*	*p*	*B*	*SE*	*p*	*B*	*SE*	*p*
X: Experimental Condition	−0.59	0.03	<0.001	0.36	0.03	<0.001	0.03	0.04	0.528
W_1_: Narcissistic Admiration (ADM)	0.03	0.03	0.241	0.04	0.03	0.265	0.11	0.04	0.002
W_2_: Narcissistic Rivalry (RIV)	0.02	0.03	0.406	0.06	0.03	0.092	−0.20	0.04	<0.001
M_1_: Perceived Recognition	–	–	–	–	–	–	0.29	0.05	<0.001
M_2_: Perceived Freedom Threat	–	–	–	–	–	–	0.21	0.04	<0.001
X × W_1_: Condition × ADM	−0.02	0.03	0.427	0.03	0.03	0.265	0.02	0.03	0.608
X × W_2_: Condition × RIV	0.02	0.03	0.406	−0.01	0.03	0.805	−0.07	0.04	0.063
Constant	−0.02	0.03	0.597	0.01	0.03	0.788	0.00	0.03	0.972
	*R*^2^ = 0.35			*R*^2^ = 0.13			*R*^2^ = 0.09		
	*F* = 82.06, *p* < 0.001			*F* = 23.76, *p* < 0.001			*F* = 11.46, *p* < 0.001		
Conditional Indirect Association of Experimental Condition with Recognition-Based Preferences through Perceived Recognition									
Condition	*Coeff.*		*Boot SE*		*Boot LCI*		*Boot UCI*		
Low Narcissistic Admiration	−0.16		0.03		−0.23		−0.10		
High Narcissistic Admiration	−0.18		0.04		−0.25		−0.11		
Low Narcissistic Rivalry	−0.18		0.04		−0.26		−0.11		
High Narcissistic Rivalry	−0.16		0.03		−0.22		−0.10		
Conditional Indirect Association of Experimental Condition with Recognition-Based Preferences through Perceived Freedom Threat									
Condition	*Coeff.*		*Boot SE*		*Boot LCI*		*Boot UCI*		
Low Narcissistic Admiration	0.07		0.02		0.04		0.11		
High Narcissistic Admiration	0.08		0.02		0.04		0.13		
Low Narcissistic Rivalry	0.08		0.02		0.04		0.12		
High Narcissistic Rivalry	0.07		0.02		0.04		0.11		

### 3.8. Entitlement-Based Preferences

The results of the conditional process analysis predicting entitlement-based preferences are presented in Table 8. Experimental condition was not associated with entitlement-based preferences (*B* = −0.01, *CI*_95%_[−0.10, 0.07], *SE* = 0.04, *t* = −0.33, *p* = 0.744). Both narcissistic admiration (*B* = 0.12, *CI*_95%_[0.06, 0.19], *SE* = 0.03, *t* = 3.57, *p* < 0.001) and narcissistic rivalry (*B* = 0.17, *CI*_95%_[0.11, 0.24], *SE* = 0.03, *t* = 5.01, *p* < 0.001) were positively associated with entitlement-based preferences. However, neither narcissistic admiration (*B* = −0.01, *CI*_95%_[−0.07, 0.06], *SE* = 0.03, *t* = −0.16, *p* = 0.871) nor narcissistic rivalry (*B* = −0.03, *CI*_95%_[−0.10, 0.04], *SE* = 0.03, *t* = −0.81, *p* = 0.416) moderated the effect of experimental condition on entitlement-based preferences. With respect to the mediators, perceived recognition was negatively associated with entitlement-based preferences (*B* = −0.17, *CI*_95%_[−0.27, −0.07], *SE* = 0.05, *t* = −3.48, *p* < 0.001), whereas perceived freedom threat was not associated with entitlement-based preferences (*B* = 0.07, *CI*_95%_[−0.01, 0.16], *SE* = 0.04, *t* = 1.73, *p* = 0.085).

Experimental condition also demonstrated significant indirect associations with entitlement-based preferences through both mediators. Specifically, condition had a positive indirect effect via perceived recognition (*B* = 0.08, *CI*_95%_[0.02, 0.14], *SE* = 0.03, *z* = 2.72, *p* = 0.007). However, this indirect association was not moderated by narcissistic admiration (*B* = 0.00, *CI*_95%_[−0.01, 0.02], *SE* = 0.01) or narcissistic rivalry (*B* = −0.01, *CI*_95%_[−0.02, 0.00], *SE* = 0.01).

Similarly, experimental condition had a positive indirect association with entitlement-based preferences through perceived freedom threat (*B* = 0.04, *CI*_95%_[0.01, 0.07], *SE* = 0.02, *z* = 2.40, *p* = 0.016). Again, this indirect association was not moderated by narcissistic admiration (*B* = 0.00, *CI*_95%_[−0.00, 0.01], *SE* = 0.01) or narcissistic rivalry (*B* = 0.00, *CI*_95%_[−0.01, 0.01], *SE* = 0.01).

**Table 8:** Results of the conditional process analysis for entitlement-based preferences.

	Outcome								
	M_1_: Perceived Recognition			M_2_: Perceived Freedom Threat			Y: Entitlement-Based Preferences		
Predictor	*B*	*SE*	*p*	*B*	*SE*	*p*	*B*	*SE*	*p*
X: Experimental Condition	−0.59	0.03	<0.001	0.36	0.03	<0.001	−0.01	0.04	0.744
W_1_: Narcissistic Admiration (ADM)	0.03	0.03	0.241	0.04	0.03	0.265	0.12	0.03	<0.001
W_2_: Narcissistic Rivalry (RIV)	0.02	0.03	0.406	0.06	0.03	0.092	0.17	0.03	<0.001
M_1_: Perceived Recognition	–	–	–	–	–	–	–0.17	0.05	<0.001
M_2_: Perceived Freedom Threat	–	–	–	–	–	–	0.07	0.04	0.085
X × W_1_: Condition × ADM	−0.02	0.03	0.427	0.03	0.03	0.265	−0.01	0.03	0.871
X × W_2_: Condition × RIV	0.02	0.03	0.406	−0.01	0.03	0.805	−0.03	0.03	0.416
Constant	−0.02	0.03	0.597	0.01	0.03	0.788	0.00	0.03	0.999
	*R*^2^ = 0.35			*R*^2^ = 0.13			*R*^2^ = 0.10		
	*F* = 82.06, *p* < 0.001			*F* = 23.76, *p* < 0.001			*F* = 12.29, *p* < 0.001		
Conditional Indirect Association of Experimental Condition with Entitlement-Based Preferences through Perceived Recognition									
Condition	*Coeff.*		*Boot SE*		*Boot LCI*		*Boot UCI*		
Low Narcissistic Admiration	0.10		0.03		0.04		0.16		
High Narcissistic Admiration	0.10		0.03		0.05		0.17		
Low Narcissistic Rivalry	0.11		0.03		0.05		0.17		
High Narcissistic Rivalry	0.09		0.03		0.04		0.16		
Conditional Indirect Association of Experimental Condition with Entitlement-Based Preferences through Perceived Freedom Threat									
Condition	*Coeff.*		*Boot SE*		*Boot LCI*		*Boot UCI*		
Low Narcissistic Admiration	0.02		0.02		0.00		0.05		
High Narcissistic Admiration	0.03		0.02		−0.01		0.07		
Low Narcissistic Rivalry	0.03		0.02		−0.01		0.06		
High Narcissistic Rivalry	0.03		0.02		−0.01		0.06		

Across all outcome models, the indirect effects of norm framing through perceived recognition and perceived freedom threat were consistent and statistically robust. Importantly, these indirect pathways were not conditioned by levels of narcissistic admiration or narcissistic rivalry. In other words, the appraisal-based mechanisms linking norm framing to reactance-related, affective, evaluative, and behavioral responses operated similarly across individual differences in narcissistic self-regulation. This pattern suggests that the situational meaning of norm framing exerted broadly similar effects on proximal appraisals and immediate responses across the admiration and rivalry dimensions assessed here.

### 3.9. Sensitivity Analysis Including Excluded Participants

To evaluate the robustness of the findings, we re-estimated all primary conditional process models using the full sample (*N* = 874), including participants who were excluded from the primary analyses based on the predefined data-quality criteria (i.e., univariate outliers, highly inconsistent response patterns, and straightlining). The overall pattern of results remained substantively identical. Experimental condition continued to show significant associations with perceived recognition and perceived freedom threat, and both mediators retained their significant associations with all focal outcomes. The indirect effects via perceived recognition and perceived freedom threat remained statistically significant across outcomes, and no additional significant interaction effects involving narcissistic admiration or narcissistic rivalry emerged. Parameter estimates were highly comparable in magnitude and direction to those obtained in the primary analytic sample (*N* = 776). These findings indicate that the results are robust to analytic sample specification and are not attributable to the applied exclusion criteria.

## 4. Discussion

### 4.1. Summary of the Findings

Overall, the results supported the predicted condition effects on perceived recognition and perceived freedom threat, as well as the corresponding indirect effects on the affective-evaluative outcomes. At the same time, the behavioral-preference findings were more mixed, and the hypothesized moderation effects involving narcissistic admiration and narcissistic rivalry were not supported. Thus, the clearest contribution of the study lies in identifying an immediate appraisal-based pathway through which recognition-based versus status-challenging norm framing shaped short-term responses in a routine public parenting context.

The present study shows that even subtle shifts in how parents frame everyday norms can reliably activate appraisal mechanisms that shape emotional, cognitive, and behavioral reactions in public settings. The findings support a clear appraisal-based pathway in which recognition-based framing increased perceived recognition and reduced perceived freedom threat, whereas status-challenging framing reduced perceived recognition and elevated perceived freedom threat. These appraisal differences predicted state reactance, negative affect, evaluations of the initiating parent, and behavioral preferences. However, the behavioral-preference outcomes were more mixed than the affective-evaluative outcomes, indicating that self-reported action tendencies may be shaped by a more complex combination of recognition-related and autonomy-related motives.

At the same time, the pattern of indirect effects suggests that the recognition pathway was often stronger than the perceived freedom-threat pathway, indicating that the two appraisal channels were not symmetrical in their downstream weight. The consistency of these indirect effects across the admiration and rivalry dimensions assessed here underscores the importance of situational meaning as an immediate determinant of autonomy-relevant responses. At the same time, this pattern should not be interpreted as evidence that personality is unimportant more broadly, but only that the appraisal effects of the manipulation were not contingent on the specific grandiose narcissism dimensions measured in the present study.

Recognition-based framing substantially increased perceived recognition and reduced perceived freedom threat compared to status-challenging framing. This pattern aligns with identity-based interactionist perspectives showing that subtle signals of legitimacy and fairness shape situational appraisals in role-relevant contexts [15,16]. It is also consistent with recognition-centered models of narcissistic self-regulation that emphasize the motivational importance of being acknowledged and treated as legitimate within social interactions [1,3]. The magnitude and consistency of these findings indicate that the social meaning embedded in norm enforcement, even in the absence of overt hostility or escalation, is sufficient to shift autonomy-relevant appraisals in everyday public parenting contexts.

Perceived recognition and perceived freedom threat operated as parallel mediators, indicating that at least two distinct appraisal pathways link norm framing to emotional, cognitive, and behavioral responses. This dual-path structure meaningfully extends psychological reactance theory by demonstrating that reactance-related mechanisms apply not only to explicit persuasion attempts but also to ordinary interpersonal norm enforcement. Classical and contemporary accounts of reactance emphasize perceived freedom threat as the proximal trigger of motivational resistance [22,34], and recent meta-analytic findings support its reliability across communication contexts [8,24]. The present results broaden this framework by showing that similar appraisal dynamics emerge in brief, low-intensity parenting interactions that are socially ordinary and non-escalated. This highlights the ecological relevance of appraisal-based reactance processes in everyday community settings.

Contrary to expectations derived from dynamic models of narcissism, neither narcissistic admiration nor rivalry moderated the indirect pathways from experimental condition to outcomes through the appraisal layer. This does not imply that personality is unimportant, but rather that the framing manipulation exerted a broadly similar influence on appraisal processes across the specific grandiose narcissism dimensions assessed here. At the same time, narcissistic rivalry showed consistent direct associations with reactance-related and entitlement-based responding. This pattern aligns with the defensive status-protection pathway described in the Narcissistic Admiration and Rivalry Concept [1] and with process models of narcissistic status pursuit that highlight antagonistic responding when threat is perceived [2]. In subtle, low-intensity public interactions, narcissistic rivalry may therefore function less as a perceptual amplifier of situational cues and more as a stable response orientation that shapes downstream reactions once appraisals have occurred. Accordingly, the moderated mediation component of the model was not supported in the manner originally hypothesized, and the null moderation findings point to a more limited role for the measured grandiose narcissism dimensions of narcissistic admiration and rivalry in shaping the appraisal effects of the manipulation.

Importantly, the absence of moderation by narcissistic admiration and rivalry should be interpreted cautiously. The norm-framing manipulation appeared to function as a relatively clear situational cue for immediate appraisals within the present design, but this conclusion is limited to the admiration and rivalry dimensions of grandiose narcissism assessed here. It should not be generalized to vulnerable narcissism or to personality processes beyond narcissism more broadly. Thus, the present findings are more appropriately interpreted as evidence that the appraisal effects of the manipulation were not conditional on the specific grandiose narcissism dimensions measured in this study.

The magnitude of the experimental effects warrants careful interpretation. The manipulation was intentionally designed to depict a normatively ordinary, non-escalated interaction. However, the shift in normative meaning—from mutual legitimacy to implied hierarchy—may have functioned as a psychologically diagnostic cue within a role-relevant context. In identity-salient situations such as public parenting encounters, even subtle variations in norm framing may be rapidly appraised as signals of inclusion versus diminished standing.

Accordingly, the observed effects likely reflect the manipulation’s impact on perceived legitimacy and autonomy rather than any increase in overt emotional intensity or interpersonal conflict. That is, the potency of the framing shift appears to have operated through appraisal processes tied to identity and standing, rather than through escalation of affective arousal per se.

### 4.2. Appraisal Processes in Public Parenting Contexts

The results reinforce the theoretical distinction between legitimacy affirmation and autonomy threat. Recognition did not merely reflect the absence of threat but instead may have captured the perception of being respected and treated as a legitimate participant within a shared normative space. Its strong associations with evaluations and behavioral preferences highlight the importance of recognition processes in socially visible parenting roles. The findings further indicate that recognition and perceived freedom threat should not be viewed as opposite ends of a single evaluative continuum. Although negatively correlated, they operate as distinct appraisal channels with partially independent downstream implications: recognition reflects perceived legitimacy and inclusion, whereas perceived freedom threat reflects perceived restriction of agency and autonomy. Demonstrating that both processes operate simultaneously strengthens appraisal-based models of social interaction by clarifying that legitimacy affirmation and autonomy threat represent conceptually and functionally separable mechanisms.

The relative magnitude of the indirect effects further suggests that these two appraisal channels did not contribute equally in the present design. Across several outcomes, the indirect pathway through perceived recognition was larger than the pathway through perceived freedom threat, which raises the possibility that variation in recognition was especially central to participants’ responses. Thus, the present manipulation should not be interpreted as establishing recognition and perceived freedom threat as bipolar opposites. Rather, the status-challenging framing appears to have combined reduced recognition with increased freedom threat, with recognition-related appraisals carrying particularly strong downstream consequences in the current data. Future research should disentangle these components more directly by varying the degree of recognition more systematically and, ideally, by manipulating recognition-relevant and freedom-threatening cues more independently.

The pattern across outcome variables also suggests that they should not be treated as fully redundant. The clearest and most consistent effects emerged for affective-evaluative responses such as state reactance, negative affect, and evaluations of the initiating parent, whereas the behavioral preference indices were more differentiated. For example, recognition-based preferences did not show a direct condition effect, and entitlement-based preferences showed a weaker and less consistent pattern than the affective-evaluative outcomes. This suggests that subtle norm framing may more readily shape immediate appraisals and affective-evaluative reactions than self-reported action tendencies, which may remain more differentiated and partly shaped by distinct motivational considerations.

One particularly noteworthy result was that perceived freedom threat was positively, rather than negatively, associated with recognition-based preferences. This pattern did not conform to our original directional expectation and suggests that recognition-based preferences may not reflect only low-threat or affiliative responding. In everyday public parenting encounters, heightened autonomy threat may sometimes motivate attempts to restore order, legitimacy, or mutual coordination through prosocial or norm-consistent responses rather than through overt opposition alone. Put differently, recognition-based preferences may in some cases function as a regulation strategy under tension, not merely as an indicator of feeling recognized. This interpretation remains tentative, but it may help explain why the behavioral-preference findings were more mixed than the affective-evaluative outcomes and underscores the potential value of distinguishing immediate appraisals from self-reported action tendencies. In addition, the mixed pattern for the behavioral-preference outcomes suggests that these indices may reflect multiple motivational tendencies simultaneously, and future research should examine these response options with more differentiated behavioral measures.

Identity theory posits that discrepancies between role meanings and social feedback shape affective and behavioral outcomes [15,16]. Parenting, as a highly norm-regulated role, becomes especially sensitive to legitimacy cues when enacted in public settings where behavior is visible and socially evaluated. Recent work linking parental identity to parental well-being underscores the importance of receiving feedback that aligns with role expectations [19]. The present findings extend this perspective by showing that even brief, routine interactions involving shared resources can trigger identity-relevant appraisals, highlighting how subtle variations in norm framing may affirm or threaten parents’ sense of legitimacy in public spaces.

### 4.3. Public Health Implications

At the level of direct evidence, the present study shows that a single instance of norm framing in a playground encounter can alter perceived recognition and perceived freedom threat, which, in turn, shape immediate affective, evaluative, and behavioral responses. This is the primary empirical contribution of the study.

These findings may also be relevant to broader public health perspectives on parental stress, insofar as everyday family environments involve recurring interpersonal experiences [4,5,6,7,10,11]. However, the present design does not test cumulative stress processes, long-term outcomes, or intervention effects directly. Therefore, the broader public health implications should be understood as theoretically informed directions for future longitudinal and intervention-based research rather than as direct conclusions of the present study.

Recognition-based communication reduced perceived freedom threat and subsequent reactance, suggesting that interactional framing in shared family spaces may be relevant to future research on parenting stress and everyday family environments. However, the present findings do not establish long-term effects, broader mental health outcomes, or population-level intervention value. Rather, they identify an immediate appraisal-based mechanism that may serve as a useful starting point for future longitudinal, diary-based, and intervention-oriented research.

### 4.4. Limitations

Several limitations qualify the interpretation of these findings. First, the study employed a single-exposure experimental design. Although this approach strengthens internal validity, it does not directly assess cumulative stress processes, which are central to the public health framing of parenting stress. The present design captures immediate appraisal and response dynamics rather than chronic outcomes, and therefore cannot determine whether repeated recognition-based or status-challenging encounters contribute to sustained psychological strain, burnout, or physiological stress markers. The assumption that such micro-level exposures accumulate over time is theoretically grounded but remains empirically untested in this context. Longitudinal or diary-based methodologies will be necessary to evaluate whether repeated activation of perceived freedom threat or diminished recognition predicts enduring changes in parental well-being. Accordingly, any link between the present findings and longer-term parental stress should be interpreted as a theoretical extension rather than an empirical conclusion of this study. For the same reason, the present findings should not be interpreted as evidence for population-level intervention effects or broader mental health outcomes beyond the immediate responses assessed here.

Second, all outcomes were self-reported and measured immediately after exposure. Although this temporal proximity reduces retrospection bias, it limits the ability to assess observable behavioral consequences or longer-term relational outcomes that may emerge following repeated interactions. Incorporating behavioral observations, ecological momentary assessment, or physiological indicators in future research would strengthen external validity and provide a more comprehensive understanding of how appraisal processes translate into real-world behavioral and relational patterns.

Third, although the video stimuli were rigorously standardized and validated by expert ratings, they were AI generated. Even with careful production controls, subtle perceptual variations may persist, raising questions about how closely these stimuli approximate naturally occurring interactions. Replication using live actors or naturalistic field manipulations would strengthen confidence in ecological generalizability and help determine whether appraisal patterns observed here extend to fully naturalistic encounters.

Fourth, the sample consisted of Israeli parents within a specific developmental window. Cultural norms regarding authority, negotiation, and parental legitimacy vary substantially across societies, and these differences may shape how recognition-based versus status-challenging framing is interpreted in public settings. Variation in tolerance for hierarchical assertion, expectations regarding turn-taking, and norms surrounding parental behavior in shared spaces could influence both the magnitude and possibly the direction of appraisal effects across cultural contexts. Consequently, cross-cultural replication is necessary to determine whether the mechanisms identified here generalize across differing normative climates and levels of power distance. Comparative designs would help specify the boundary conditions of the present appraisal-based model and clarify how culturally embedded expectations shape responses to everyday norm enforcement.

Fifth, the design focused on trait-level narcissistic admiration and rivalry and therefore assessed only dimensions of grandiose narcissism. The study did not include vulnerable narcissism or other potentially relevant dispositional constructs such as trait reactance or social dominance orientation. As a result, the absence of moderation should not be interpreted as evidence that personality is unimportant for responses to everyday norm framing more generally. Expanding the range of measured traits in future research would provide a clearer understanding of how different personality-based factors intersect with situational framing to shape appraisal and response patterns. This limitation is also relevant to the applied implications of the study, because the feasibility and effectiveness of context-focused norm interventions may depend on broader motivational and dispositional factors not assessed here.

Finally, although perceived freedom threat was directly assessed, status or legitimacy threat was inferred conceptually rather than measured as a distinct construct. Moreover, the two-condition design did not allow recognition-related and freedom-threatening aspects of the interaction to be varied independently or across graded levels. More explicit measurement of status or legitimacy threat, together with more differentiated manipulations, would allow future studies to determine whether diminished recognition, status-related threat, and perceived freedom threat exert independent or interacting effects on downstream responses. Clarifying these distinctions would refine the appraisal architecture and improve understanding of the specific mechanisms through which norm framing shapes psychological reactions.

## 5. Conclusions

Subtle differences in norm framing within ordinary playground interactions systematically shaped perceived recognition and perceived freedom threat, which, in turn, predicted immediate affective, evaluative, and behavioral responses. Narcissistic rivalry contributed directly to reactance-related responding but did not alter the appraisal structure elicited by situational framing. These findings extend reactance theory into everyday public parenting contexts and clarify that recognition-based versus status-challenging framing can shape immediate appraisals through related but non-equivalent pathways involving perceived recognition and perceived freedom threat. By demonstrating that normatively plausible communication cues influenced both perceived recognition and perceived freedom threat, while suggesting a particularly strong role for recognition-related appraisals in the present design, the study highlights the importance of situational meaning in shaping immediate psychological responses, while leaving open the role of other personality dimensions, including vulnerable narcissism. Although the broader public health implications remain provisional, the present findings primarily identify an immediate appraisal-based mechanism linking norm framing to parents’ short-term responses in everyday encounters. Whether repeated exposure to such encounters has cumulative implications for parental stress or broader mental health outcomes remains a question for future longitudinal and intervention-based research.

## Figures and Tables

**Table 1:** Sociodemographic and background information.

		Men (*n* = 389)		Women (*n* = 387)		
		Experimental Condition				
	Total Sample (*N* = 776)	Recognition-Based (*n* = 196)	Status-Challenging (*n* = 193)	Recognition-Based (*n* = 202)	Status-Challenging (*n* = 185)	Recognition-Based ≠ Status-Challenging
Age	35.45	35.73	35.11	35.48	35.49	*t* = −1.05
Number of children	2.30	2.27	2.47	2.26	2.21	*t* = 0.93
Age of focal child	3.88	3.80	3.91	4.03	3.79	*t* = −0.43
Frequency of playground use	4.75	4.83	4.59	4.77	4.82	*t* = −0.99
Education						*χ*^2^ = 4.59
No high school degree	10.4%	13.3%	16.6%	7.4%	4.3%	
High school degree	21.5%	23.0%	23.3%	16.3%	23.8%	
Bachelor’s degree	46.0%	41.8%	46.1%	48.5%	47.6%	
Master’s degree	20.4%	19.9%	13.0%	25.7%	22.7%	
Ph.D. or equivalent	1.7%	2.0%	1.0%	2.0%	1.6%	
Employment						*χ*^2^ = 4.00
Full time	78.5%	88.8%	86.0%	70.3%	68.6%	
Part time	12.4%	6.6%	8.8%	15.8%	18.4%	
Unemployed	2.4%	2.6%	2.1%	2.5%	2.7%	
Going to school	2.8%	1.0%	1.0%	6.4%	3.8%	
Retired	1.4%	1.0%	1.6%	1.0%	2.2%	
Marital Status						*χ*^2^ = 10.31
Single	1.8%	1.5%	0.5%	3.0%	2.7%	
Dating	1.2%	0.0%	1.0%	1.0%	2.2%	
Cohabiting	3.5%	3.1%	3.1%	2.5%	5.4%	
Married	89.6%	90.3%	93.8%	88.6%	85.4%	
Separated	0.6%	1.0%	0.5%	1.0%	0.0%	
Divorced	3.2%	4.0%	1.0%	4.0%	3.8%	
Widowed	0.1%	0.0%	0.0%	0.0%	0.5%	
Household income						*χ*^2^ = 2.57
Very high	13.4%	18.9%	17.1%	6.9%	10.8%	
Somewhat high	25.9%	28.6%	30.6%	27.2%	16.8%	
Moderate	28.9%	32.7%	26.9%	26.2%	29.7%	
Somewhat low	21.1%	12.8%	16.6%	26.2%	29.2%	
Very low	10.7%	7.1%	8.8%	13.4%	13.5%	
Religiosity						*χ*^2^ = 8.77
Secular	45.4%	49.5%	40.4%	46.0%	45.4%	
Traditional	30.4%	31.1%	17.6%	39.1%	33.5%	
Religious	17.8%	17.9%	26.9%	11.4%	15.1%	
Ultra-Orthodox	6.4%	1.5%	15.0%	3.5%	5.9%	

**Table 2:** (**A**) Descriptive statistics by experimental condition. (**B**) Zero-order correlations among the study variables by experimental condition.

(**A**)									
	**Recognition-Based** **Condition (*n* = 398)**				**Status-Challenging** **Condition (*n* = 378)**				
	***M***	***SD***	***Skewness***	***Kurtosis***	***M***	***SD***	***Skewness***	***Kurtosis***	
1. Narcissistic Admiration	3.49	0.79	−0.13	−0.14	3.50	0.83	0.09	−0.10	
2. Narcissistic Rivalry	2.07	0.75	0.78	0.15	2.08	0.76	0.67	−0.20	
3. Perceived Recognition	5.10	1.47	−0.37	−0.75	3.01	1.43	0.69	0.03	
4. Perceived Freedom Threat	2.84	0.99	0.12	−0.64	3.57	0.93	−0.48	−0.12	
5. State Reactance	2.12	1.04	0.55	−0.81	3.42	0.99	−0.56	−0.11	
6. Negative Affect	2.41	1.01	0.52	−0.52	3.64	0.89	−0.60	−0.18	
7. Evaluation of the Initiating Parent	5.11	1.66	−0.60	−0.46	2.81	1.54	0.79	0.21	
8. Recognition-Based Preferences	4.28	0.81	−0.85	−0.16	4.16	0.84	−0.77	−0.30	
9. Entitlement-Based Preferences	2.74	0.95	0.21	−0.34	2.96	0.99	0.06	−0.44	
(**B**)									
	**1**	**2**	**3**	**4**	**5**	**6**	**7**	**8**	**9**
1. Narcissistic Admiration	–	0.14 **	0.03	0.09	0.11 *	0.04	−0.08	0.12 *	0.14 **
2. Narcissistic Rivalry	0.21 ***	–	0.09	0.07	0.12 *	0.03	0.08	−0.21 ***	0.16 **
3. Perceived Recognition	0.06	−0.01	–	−0.47 ***	−0.59 ***	−0.58 ***	0.72 ***	−0.08	−0.16 **
4. Perceived Freedom Threat	0.02	0.07	−0.52 ***	–	0.68 ***	0.58 ***	−0.49 ***	0.31 ***	0.11 *
5. State Reactance	0.07	0.16 ***	−0.72 ***	0.64 ***	–	0.80 ***	−0.64 ***	0.22 ***	0.25 ***
6. Negative Affect	−0.02	0.09	−0.72 ***	0.58 ***	0.82 ***	–	−0.63 ***	0.22 ***	0.15 **
7. Evaluation of the Initiating Parent	0.02	−0.10	0.67 ***	−0.45 ***	−0.69 ***	−0.69 ***	–	−0.10	−0.13 *
8. Recognition-Based Preferences	0.09	−0.10 *	0.33 ***	−0.14 **	−0.27 ***	−0.25 ***	0.30 ***	–	0.19 ***
9. Entitlement-Based Preferences	0.17 ***	0.24 ***	−0.17 ***	0.20 ***	0.33 ***	0.26 ***	−0.20 ***	−0.01	–

Note. Recognition-based and status-challenging condition statistics are presented separately for each study variable. *M* = mean; *SD* = standard deviation. Note. The values below the diagonal are taken from participants in the recognition-based condition, whereas the values above the diagonal are taken from participants in the status-challenging condition. * *p* < 0.05; ** *p* < 0.01; *** *p* < 0.001.

## Data Availability

To enhance transparency and reproducibility, the study data and materials are openly available on the Open Science Framework (OSF) at https://osf.io/9uemc, accessed on 21 April 2026. The repository includes the finalized stimulus videos, the full dialogue scripts, storyboard prompts, production specifications, post-processing documentation, and the anonymized SPSS data file used for the reported analyses.
